# Combined pangenomics and transcriptomics reveals core and redundant virulence processes in a rapidly evolving fungal plant pathogen

**DOI:** 10.1186/s12915-023-01520-6

**Published:** 2023-02-06

**Authors:** Hongxin Chen, Robert King, Dan Smith, Carlos Bayon, Tom Ashfield, Stefano Torriani, Kostya Kanyuka, Kim Hammond-Kosack, Stephane Bieri, Jason Rudd

**Affiliations:** 1 Department of Protecting Crops and the Environment, Rothamsted Research, Harpenden, Herts UK; 2 Present address: School of Agriculture, Shenzhen Campus of Sun Yat-sen University, Guangming District, Shenzhen, Guangdong People’s Republic of China; 3 Crop Health and Protection (CHaP), Rothamsted Research, Harpenden, Herts UK; 4 Syngenta Crop Protection AG, Schaffhauserstrasse 101, CH-4332 Stein, Switzerland; 5 Present address: National Institute for Agricultural Botany (NIAB), 93 Lawrence Weaver Road, Cambridge, UK

**Keywords:** *Foxtail mosaic virus*, *Septoria tritici*, Necrotrophic effector, Essential genes, Accessory chromosomes, Dothideomycetes, Chromosome instability, *Mycosphaerella* spp

## Abstract

**Background:**

Studying genomic variation in rapidly evolving pathogens potentially enables identification of genes supporting their “core biology”, being present, functional and expressed by all strains or “flexible biology”, varying between strains. Genes supporting flexible biology may be considered to be “accessory”, whilst the “core” gene set is likely to be important for common features of a pathogen species biology, including virulence on all host genotypes. The wheat-pathogenic fungus *Zymoseptoria tritici* represents one of the most rapidly evolving threats to global food security and was the focus of this study.

**Results:**

We constructed a pangenome of 18 European field isolates, with 12 also subjected to RNAseq transcription profiling during infection. Combining this data, we predicted a “core” gene set comprising 9807 sequences which were (1) present in all isolates, (2) lacking inactivating polymorphisms and (3) expressed by all isolates. A large accessory genome, consisting of 45% of the total genes, was also defined. We classified genetic and genomic polymorphism at both chromosomal and individual gene scales. Proteins required for essential functions including virulence had lower-than average sequence variability amongst core genes. Both core and accessory genomes encoded many small, secreted candidate effector proteins that likely interact with plant immunity. Viral vector-mediated transient in planta overexpression of 88 candidates failed to identify any which induced leaf necrosis characteristic of disease. However, functional complementation of a non-pathogenic deletion mutant lacking five core genes demonstrated that full virulence was restored by re-introduction of the single gene exhibiting least sequence polymorphism and highest expression.

**Conclusions:**

These data support the combined use of pangenomics and transcriptomics for defining genes which represent core, and potentially exploitable, weaknesses in rapidly evolving pathogens.

**Supplementary Information:**

The online version contains supplementary material available at 10.1186/s12915-023-01520-6.

## Background

The sustainable control of infectious diseases affecting animals and plants is challenged by evolution of the causal micro-organisms [1, 2]. The most difficult to control diseases are those caused by the most rapidly evolving species, which can quickly respond to selective pressures, including natural host immunity, adverse environmental conditions and/or anti-infective drugs [1, 3]. The potential for microbial pathogens to rapidly evolve is determined by several features, including amongst others; (1) rapid life cycling and (2) mechanisms which promote sexual reproduction [4]. The latter can lead to large levels of standing genetic variation existing within pathogen populations which can be sustained and amplified in the face of external selective pressures. Whilst particular genes maybe likely to exhibit natural genetic sequence variation, driving their evolution, others cannot be lost (or inactivated by mutation) without affecting a pathogens’ fitness. For eukaryotic and prokaryotic plant pathogens, this difference in levels of polymorphisms between genes has led to the realisation of “two-speed genomes” [5], which comprise some components which rapidly evolve and are thought to respond to external drivers (host range and immunity, etc.), whilst the more slowly evolving set contains genes with core housekeeping and other essential functions. These accessory and core parts of pathogen genomes can be assembled into a “pangenome” which should represent near to the complete set of genes present in a species [6]. The size of core and accessory portions of pangenomes vary within species of bacteria, fungi and oomycetes [7–13] and may give an indication of the potential for microbial populations to evolve rapidly to selective pressures. It is conceivable that the larger the accessory pangenome is relative to the core, the more capable these populations are to rapidly evolve.

The ascomycete fungal pathogen of wheat, *Zymoseptoria tritici*, is the causal agent of Septoria tritici blotch (STB), a globally important disease which threatens food security [14]. The pathogen has also long been regarded as a model system for studies on population biology and evolution [15–18]. This is because *Z. tritici* has a high rate of sexual recombination which sustains large amounts of standing genetic diversity within its populations and allows rapid adaptation at both global and local scales [17, 19–24]. As a consequence major wheat genes conferring resistance to *Z. tritici* are rapidly overcome [25]. In addition, most of the widely used commercial fungicides lose their effectiveness over time [3]. The combination of these two factors poses a threat to global wheat production which needs to be urgently addressed. *Z. tritici* has also recently emerged as a new model for pathogen genomics with many high-quality genome references now available [26] in addition to pangenomes constructed from large numbers of individual isolate genomes [27, 28]. Perhaps one of the most interesting features of the *Z. tritici* genome is the presence of 13 core chromosomes (1-13) found in all isolates but then up to a further 8 smaller accessory chromosomes (14-21) which show presence/absence and structural polymorphism between isolates [26, 29]. Fortunately, one of the community reference isolates, and the first to be completely sequenced, the Dutch field isolate IPO323 collected in the 1980s, carries 21 chromosomes, the largest number observed to date. Therefore, IPO323 serves as an excellent benchmark for studying variation in other isolates and as a scaffold for building pangenomes.

The asexual infection cycle of *Z. tritici* is typical of many related plant pathogens affecting many food crops [30]. These fungi, belonging to the *Mycosphaerellaceae* family of the order Dothideomycetes, typically invade plant tissues through stomata [30, 31]. There then follows a period of long asymptomatic growth between plant cells, lasting at least 8 days, which ends abruptly with the formation of necrotic leaf lesions, a situation which for *Z. tritici*, appears to occur as a result of “hyperactivation” of plant immunity [32–34]. The plant cell death is subsequently associated with the formation of new sporulation structures, pycnidia, in sub-stomatal cavities and the subsequent extrusion and spread of newly formed pycnidiospores during periods of splashy rainfall. Other Dothideomycetes fungi have been shown to elicit a hyperactivation of plant immunity through the secretion of protein “effectors” which are recognised in sensitive plants, resulting in cell and tissue death (necrosis or programmed cell death). These necrotrophic fungi benefit from this response and the effector proteins involved are now referred to as necrotrophic effectors [35, 36]. Whilst there is some preliminary evidence that *Z. tritici* might also deploy necrotrophic effectors at the switch to symptomatic growth [37], invoking leaf lesions, this hypothesis has not been robustly tested to date.

The two mutually non-exclusive routes to protecting future wheat production from the impact of STB are to (1) boost natural plant disease resistance and to (2) identify new molecular targets which might be exploited for disease control. Ideally, these would enable selective control of the pathogen whilst restricting other environmental and ecological impacts to a minimum. Pathogen genomics and in particular pangenomics offer the potential to augment both strategies. Whilst proteins encoded by the accessory, rapidly evolving part of the pangenome may contain many genes which interact with plant immunity (e.g. effectors), the core genome may present targets which mutate less readily due to imparting negative fitness (and virulence) penalties. For this reason, agricultural fungicides normally target core processes, which presumably are considered less mutable. Although, as previously mentioned, even these targets can evolve to some extent to evade chemical inhibition, as described for many current fungicide target proteins [3, 14]. Most fungicides used to date have targeted quite a narrow set of molecular processes (e.g., sterol biosynthesis, respiration, etc.) [3]. There are potentially many other targets which could be used to selectively control fungal pathogens which should arise from combining and analysing multiple omics datasets. The high potential for discovery is emphasised by the fact that typically up to 40% of all genes identified in the genome sequences of pathogenic fungi (and fungi in general) are still of unknown function. Those which are present and perhaps conserved in the core genome of pathogenic fungi may be of particular relevance.

Pangenomics of rapidly evolving pathogens could be used to identify core, potentially specific gene sets, which could be exploited in future disease control [11]. The premise is that genes which are not evolving, in an otherwise rapidly evolving species, are most likely to be essential for either life or important for key virulence processes of the pathogen. Pangenomes themselves have, to date, largely been defined by sequencing genomic DNA from multiple members of a species. Whilst this is a critical and indispensable step in ascertaining the full potential of a species genomics, gene expression support is perhaps overlooked for refinement of core and accessory gene calls, particularly in relation to biological processes such as pathogenicity/virulence. For example, if a core gene, predicted through genomic DNA sequence analysis, is not expressed by a successful pathogenic strain/isolate during infection, it might be more appropriately considered accessory.

Pangenome analyses on fungi and yeasts have recently emerged and have highlighted some major differences in the size of core and accessory gene components. For example, one recent study which analysed the animal pathogenic yeasts, *Candida albicans*, *Cryptococcus neoformans*, the free-living yeast, *Saccharomyces cerevisiae* and the animal filamentous fungal pathogen *Aspergillus fumigatus*, predicted each to have > 80% genes annotated as core [10]. However, other analyses on plant-associated filamentous fungi have predicted larger accessory gene components including ~38% for the pathogens *Claviceps purpurea* [38] and ~44% in *Pyrenophora tritici-repentis* [39]. Previous comprehensive pangenomic studies on *Z. tritici* have used global and historical populations involving large isolate numbers, which have been sequenced at both fragmented and complete genome scales [27, 28]. These studies identified a large accessory gene set (~40% of all genes) for this species. In addition, this was correlated with extensive presence/absence and chromosomal structural polymorphisms, also implicating dynamic repetitive elements (including transposable elements). Further analysis also suggested that many accessory genes to be also lower expressed (on average) than core genes. These studies represent the “gold standard” for the structure of the *Z. tritici* species pangenome and the most comprehensive to date for any filamentous fungus. However, the question remains for this, and other species, as to what extent might pangenomics be used to identify core virulence genes and processes? And within the core gene sets, whether levels of population wide polymorphisms affecting amino acid changes, as well as gene expression levels, could betray the identity of key pathogen virulence genes?

In this study, we have combined genome sequencing and RNAseq-based transcriptomic approaches to produce a pangenome from a recent European collection of *Z. tritici* isolates. Our major objective was to test whether genes present within the core genome encoding proteins with low rates of amino acid polymorphism (i.e. in coding sequences) between isolates, carry important functions, including major roles in core infection processes on wheat. In agreement with the previous studies [27, 28], we demonstrate that *Z. tritici* has an extremely large accessory genome (~45% of all genes) supporting its rapidly evolving status. Furthermore, we provide biological data which clearly supports the utility of the combined use of both omics methods to identify novel core virulence genes. Conversely high-throughput viral-mediated functional protein overexpression screens failed to identify any candidate necrotrophic effectors responsible for eliciting disease lesions in wheat leaves, from either the core or accessory pangenome.

## Results

### Virulence screens on a European *Z. tritici* collection identifies similarly aggressive strains from geographically unlinked locations

**Fig. 1:**
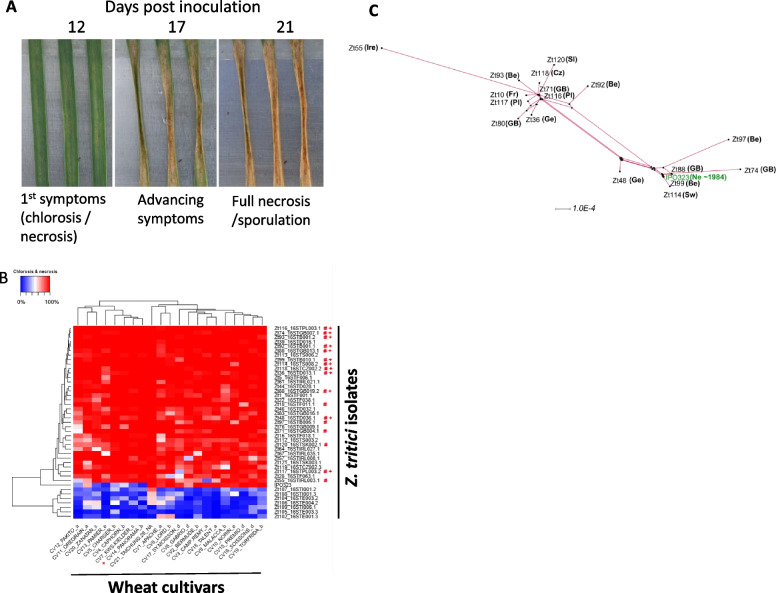
Virulence assessments of the European isolate collection against a range of hexaploid (bread) wheat cultivars. **A** Typical disease progression time course illustrating the parameters assessed in the screen, including the time taken to the appearance of first visible symptoms and for full leaf necrosis to appear in the inoculated area. The figure shows infection of wheat cultivar Riband by *Z. tritici* isolate IPO323. **B** Virulence profile of the isolates vs the cultivar panel based on levels of leaf necrosis and chlorosis. Measurements were taken by both visual assessments and by using the LemnaTec, LemnaGrid image analysis software with comparable final results. Isolates were ranked and clustered based on virulence data. *Z. tritici* isolates highlighted by # were genome sequenced to construct a pangenome. Isolates highlighted by + were also analysed by RNAseq transcriptomics. Wheat cultivar Panorama (highlighted by X) was determined to be equally and fully susceptible to most isolates and was selected as the host genotype for the leaf infection RNAseq. Note the low virulence data for the outgroup of seven isolates against all cultivars is likely to result from these isolates being adapted to causing disease on tetraploid wheat (Durum or Pasta). All data is representative of three infected leaves analysed/interaction from two biological replicate experiments (6 leaves in total). **C** SplitsTree analysis of the molecular phylogeny of isolates selected for genomic sequencing. The country of origin of isolates is shown in abbreviation (Pl = Poland; GB = Great Britain; Be = Belgium; Cz = Czech Republic; Ge = Germany; Sw = Sweden; Fr = France; Sl = Slovakia; Ir = Ireland). The reference isolate, IPO323 collected ~1984 from the Netherlands (Ne) is also represented

We tested 43 *Z. tritici* isolates for virulence on 21 broadly susceptible European bread wheat cultivars to give a total of 2709 datapoints (including three replications). Isolate information (code and country of origin) is presented in Additional file 1: Table S1. Each interaction was assessed quantitatively for the following parameters: (1) the length of the incubation period, (2) the time taken to reach complete necrosis (when it occurred) and (3) levels of asexual sporulation (Fig. 1A). Based on both computational and visual disease scoring (see Methods and Additional file 2: Fig. S1), all isolates were then grouped based on their virulence profiles on the tested cultivar panel (Fig. 1B). The positive control isolate IPO323, which serves as a global reference [26] with fully sequenced genome, was less aggressive than the majority of tested isolates against almost all cultivars. This isolate was collected in ~1984 and has been kept in storage since, with occasional re-passaging through wheat leaves; therefore, it is currently unclear whether this observation could (at least in part) have arisen from sample storage over time. We identified seven isolates which were unable to produce disease symptoms on any of the wheat cultivars (Fig. 1B). As these isolates all originated from either Spain or Italy, where tetraploid (durum/pasta) wheat (*Triticum durum* L.) is more frequently cultivated, it is likely that these isolates were durum wheat-specific [40, 41]. Significantly, isolates with similar virulence profiles did not cluster on their collection location, with isolates originating from as far east as Slovakia to as far west as Ireland all grouping together (Fig. 1B). This observation agrees well with high levels of genetic variation existing between isolates and previous studies which have demonstrated this to be as equally high on the scale of a single wheat leaf lesion, as across continents [42]. Many of the more aggressive isolates with distinct virulence profiles were subsequently selected for genomic sequencing (highlighted with “#” in Fig. 1B) and also RNAseq analysis (highlighted with “+” in Fig. 1B).

### Genome sequence and phylogenetic analysis on seventeen European isolates reveal high levels of genetic diversity

We used Illumina HiSeq 250 bp paired end read technology to assemble the gene space of seventeen new isolates (indicated by # in Fig. 1B). BUSCO analysis (core dataset Pezizomycotina) was then performed to assess the completeness of each genome assembly (Table 1). Scores > 97% in all cases indicated good assembly of the gene space (coding regions) for all isolates. A select few BUSCO genes were missing from all assemblies suggesting that *Z. tritici* may not have orthologues of these genes. To determine the amount of genetic variation affecting predicted coding regions, we then ran a SNPEff analysis for each strain against a pangenome built upon the complete genome of the reference isolate IPO323 (Table 2). This analysis demonstrated that each new strain had in excess of 160,000 SNPs against the derived pangenome. Amongst these were more than 65,000 which either caused a potential loss of protein function (high effect, frameshift, lost start, premature stop codons) or invoked an amino acid change in predicted proteins (Moderate). These data demonstrated the genetic diversity existing between the *Z. tritici* isolates.

**Table 1:** BUSCO analysis on new genomes

Genomes ID_Rothamsted	Country of origin	BUSCOs^a^			
		C [S,D]	F	M	***n***
Zt10	France	98.4% [98.3%,0.1%]	0.6%	1.0%	3156
Zt36	Germany	98.5% [98.4%,0.1%]	0.6%	0.9%	3156
Zt48	Germany	98.6% [98.5%,0.1%]	0.5%	0.9%	3156
Zt55	Ireland	98.6% [98.5%,0.1%]	0.4%	1.0%	3156
Zt71	Great Britain	98.7% [98.6%,0.1%]	0.4%	0.9%	3156
Zt74	Great Britain	98.4% [98.4%,0.0%]	0.5%	1.1%	3156
Zt80	Great Britain	98.4% [98.3%,0.1%]	0.7%	0.9%	3156
Zt88	Great Britain	98.5% [98.4%,0.1%]	0.6%	0.9%	3156
Zt92	Belgium	98.5% [98.4%,0.1%]	0.5%	1.0%	3156
Zt93	Belgium	98.5% [98.4%,0.1%]	0.6%	0.9%	3156
Zt97	Belgium	98.8% [98.7%,0.1%]	0.4%	0.8%	3156
Zt99	Belgium	97.4% [97.3%,0.1%]	1.5%	1.1%	3156
Zt114	Sweden	98.5% [98.4%,0.1%]	0.4%	1.1%	3156
Zt116	Poland	98.6% [98.6%,0.0%]	0.3%	1.1%	3156
Zt117	Poland	98.3% [98.3%,0.0%]	0.5%	1.2%	3156
Zt118	Czech Republic	98.6% [98.5%,0.1%]	0.4%	1.0%	3156
Zt120	Slovakia	98.4% [98.3%,0.1%]	0.6%	1.0%	3156

**Table 2:** Summary of SNP effect impacts for each new genome versus the IPO323 reference gene models

Genomes ID_Rothamsted	Effect impact						Total
	High	Percentage	Moderate	Percentage	Low	Percentage	
Zt10	6815	4.1%	58739	35.5%	99990	60.4%	165544
Zt36	7399	4.4%	60053	36.0%	99540	59.6%	166992
Zt48	7078	4.2%	59965	35.8%	100606	60.0%	167649
Zt55	7138	4.4%	58214	36.2%	95649	59.4%	161001
Zt71	7562	4.4%	62825	36.6%	101147	59.0%	171534
Zt74	7316	4.4%	59848	36.0%	98888	59.6%	166052
Zt80	7284	4.3%	59782	35.7%	100445	60.0%	167511
Zt88	6366	4.0%	56121	34.9%	98214	61.1%	160701
Zt92	6935	4.2%	58167	35.5%	98688	60.3%	163790
Zt93	6792	4.2%	56879	35.0%	99024	60.9%	162695
Zt97	7882	4.5%	63045	36.3%	102840	59.2%	173767
Zt99	7391	4.5%	58866	35.6%	99195	60.0%	165452
Zt114	6995	4.2%	59916	35.7%	100932	60.1%	167843
Zt116	7505	4.4%	61918	36.2%	101548	59.4%	170971
Zt117	7358	4.4%	59680	35.7%	100190	59.9%	167228
Zt118	7361	4.3%	61412	36.2%	100901	59.5%	169674
Zt120	6612	4.1%	57623	35.3%	98796	60.6%	163031

A splitstree analysis was performed on the 17 newly sequenced strains and IPO323 to determine whether isolates with similar cultivar infection patterns had any phylogenetic relationship and whether this was associated with the country of origin. Phylogeny was determined using a concatenation of six coding and non-coding sequences (see Methods and Additional file 3: Data S1). The tree (Fig. 1C) illustrates once again that there was no clear linkage between virulence profiles and sampling location for the sequenced isolates, with closely related virulence profiles (compare with Fig. 1B) seen for isolates collected from different European countries which were nevertheless phylogenetically closely related.

### Pangenome construction and analysis predicts 9807 “core” and 8083 “accessory” genes

**Fig. 2:**
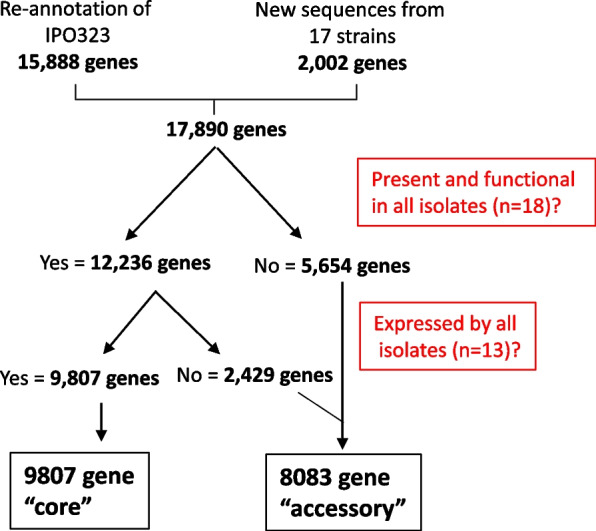
Summary of the steps used to generate the “core” and “accessory” gene calls and numbers for the newly constructed European *Z. tritici* pangenome. The numbers represent the number of genes identified at each stage in the pipeline

In addition to the genome sequences, we selected 12 isolates for RNAseq-based transcription profiling (indicated by + in Fig. 1B). Each isolate was profiled in triplicate during axenic growth in YPD broth and also at two independent stages of leaf infection; 6 days post inoculation (dpi) representing the mid symptomless phase and 9 dpi, at the transitional phase of early symptom development. The universally susceptible wheat cultivar Panorama was used for infection assays, which all isolates were able to fully infect with indistinguishable kinetics. Using this transcriptome data combined with the new genome assemblies, and the original reference sequence of IPO323 as the scaffold, we determined a pangenome for the European isolates also incorporating sequences present in other isolates which were not detected in IPO323. The steps in the pangenome construction and our criteria for categorising the genes as “core” or “accessory” are shown in Fig. 2. Importantly, inactivating (high effect, loss of function—LoF) sequence polymorphism, and presence and absence, considerations were also backed up by evidence of gene expression. Thereby a gene should be present in a functional form and expressed by all isolates, to be assigned to the “core” category. Our overall approach (Fig. 2) defined a total pangenome of 17,890 genes including 2017 which were not present in IPO323. The subsequent filtering steps categorised 9807 to reside in the core genome, with only a slightly smaller number, 8083, in the accessory set. The latter represents ~45% of the total pangenome, a slight increase on that previously described [28]. Whilst these numbers generally agree well, estimations of accessory genes are more subjective to bias due to the methods used (Panseq etc), and we cannot assume that additional future analysis may not reduce the accessory gene number using, for example, orthology-based approaches.

All pangenes were analysed for multiple features (predictions of secretion, membrane association, localisation, cysteine content) and predicted functions (BLAST, GO and Interpro annotations). A table providing all available information on all genes is provided (Additional file 4: Table S2). The table also displays the relative mean RNAseq-derived gene expression levels determined for all genes for all 12 isolates under the three conditions tested. Collectively, when also compared with the previous pangenomic studies on this organism [27, 28], it is clear that *Z. tritici* has one of the largest number of accessory genetic components of fungi studied to date, which likely reflects its rapidly evolving nature.

### Structural features of variation in the European *Z. tritici* pangenome

The goal of this study was to test the hypothesis that pangenomics could be exploited to identify important new genes, either essential for life or for virulence in pathogenic organisms. We hypothesised that these would be genes present and functional in, and expressed by, every strain of the pathogen. We further speculated that these genes would have lower levels of high and moderate-effect SNP mutations affecting coding sequences (less polymorphic) and that they may cluster in regions on particular chromosomes.

**Fig. 3:**
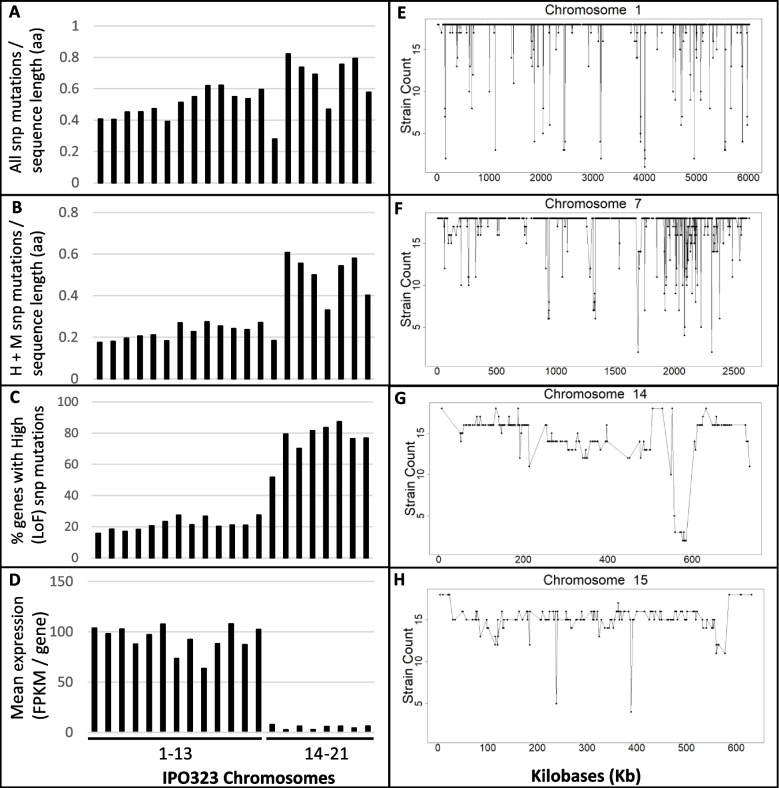
Pangenome structural features and presence/absence polymorphisms across the 21 chromosomes of reference isolate IPO323. **A** Total number of cumulative SNP mutations conferring modifier, moderate (M) and high (H—loss of function—LoF) impact changes expressed as a feature of the average protein length (aa) per chromosome. **B** Total number of cumulative SNP mutations conferring moderate and high impact changes expressed as a feature of the average protein length per chromosome. **C** Total number of cumulative SNP mutations conferring High impact changes expressed as a feature of the average protein length per chromosome. **D** Mean average expression of all genes present on each of the 21 chromosomes across all isolates. **E** Presence and absence (PaV) polymorphism of the genes predicted on core chromosome 1 of isolate IPO323 in the 17 newly sequenced isolates. **F** PaV for genes on core chromosome 7. **G** PaV for genes on accessory chromosome 15. **G**–**F** PaV for genes on accessory chromosome 15. The data highlight extensive regional variation and mark a clear distinction between the levels of overall sequence polymorphisms and presence/absence evident on the core and accessory chromosomes

We used SNP variation data (high, moderate and modifier—see Additional file 4: Table S2), as well as mean expression data for all genes, to investigate average levels of coding polymorphism and expression on a chromosomal basis, using the 21 chromosomes of IPO323 as the scaffold. Modifier SNPs represent nucleotide changes which do not invoke an amino acid change. Figure 3A demonstrates that there existed no particular bias for mutation frequency when cumulative high, moderate and modifier SNP’s were expressed as a feature of average protein length per entire chromosome. However, when modifier mutations, i.e. those which do not cause an amino acid change, were omitted, it was clear that the smallest 8 chromosomes possessed genes with higher numbers of high and moderate SNP mutations (Fig. 3B). This effect was further emphasised when only high-effect SNP’s were analysed (Fig. 3C). In contrast to the levels evident on core chromosomes 1–13 (~20%), up to 90% of genes residing on the eight smallest chromosomes 14–21 were subject to high effect, likely loss-of-function mutations in at least one isolate (Fig. 3C). The cumulative transcription data from all 12 new isolates also demonstrated relatively low average expression of genes present on chromosomes 14–21 (Fig. 3D), as had previously been observed for IPO323 [32, 43, 44]. Taken together, this data indicates a clear genomic compartmentalization, with the smallest eight accessory chromosomes harbouring sequences which are highly polymorphic, accessory and generally poorly expressed.

Comparing all genes in either the core or accessory genome revealed that ~60% of all genes in the latter were found in at least one strain with a high effect (loss of function (LoF)) mutation and that 47% were also absent from at least one strain (Additional file 4: Table S2). Similar to the analysis on the accessory chromosomes, individual accessory genes were also very poorly expressed overall across the isolates tested (Additional file 4: Table S2) and had a far higher number of sequences with unknown functions (approx. 80%). These observations compare well with previous pan-genomic studies performed on global collections of *Z. tritici* collected decades apart [27, 28].

We also performed a gene-by-gene presence and absence variation (PAV) analysis for all genes predicted in the reference isolate IPO323, for the other 17 sequenced isolates. This presence/absence data was plotted positionally for each gene across each of the 21 chromosomes present in IPO323. Figure 3E and F show data from representative core chromosomes (1 and 7), whilst Fig. 3G and H show data for representative accessory chromosomes (13 and 14). Data for the remaining chromosomes are shown in Additional file 2: Fig. S2. This analysis identified many genes present in IPO323 which are missing in a number of the re-sequenced isolates and clearly shows the difference in overall gene presence/absence between the “core” chromosomes of IPO323 (which are found in every *Z. tritici* strain) compared to the 8 smallest “accessory” chromosomes. The data also highlights a region on chromosome 7 in particular, between 1.7 Mb and 2.5 Mb, which displays a high frequency of gene absence in the new isolates (Fig. 3F). Interestingly, this region matches exactly to a genome location previously seen to have minimal or no gene expression in IPO323 either during growth in culture or during any phase of plant infection [32, 43]. This region also matches a chromosomal deletion, which was observed in a Yemeni isolate in a previous pangenome study [28]. Together, these data re-enforce the notion that *Z. tritici* represents a rapidly evolving organism with high levels, and different types, of genetic and genomic diversity within its population.

### Protein localisation predictions reveal genes in the accessory pangenome that may function in adaptation to changing environments

**Fig. 4:**
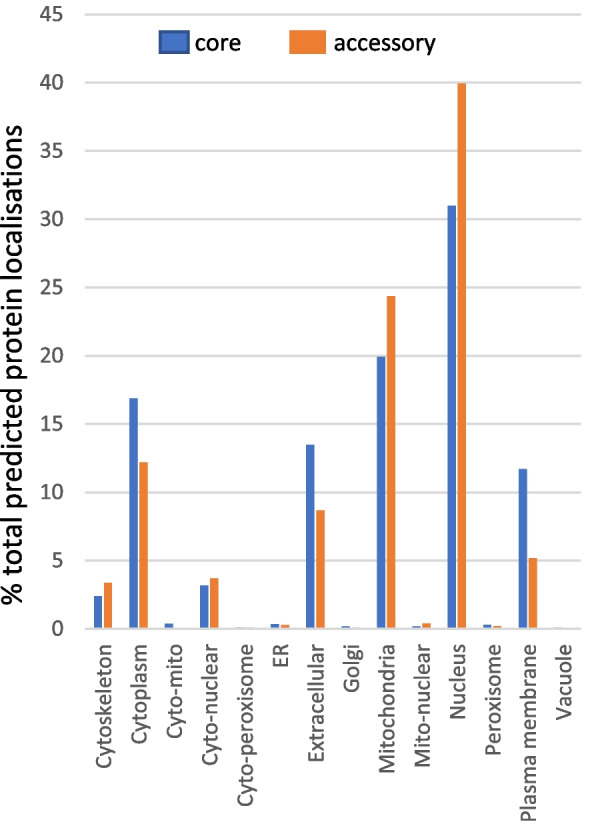
Predicted localisation of proteins encoded by core and accessory genes. Summary of percentage predicted protein localisations determined by WolfPsort for categories indicated relative to the total proteins present in the core and accessory pangenome

**Fig. 5:**
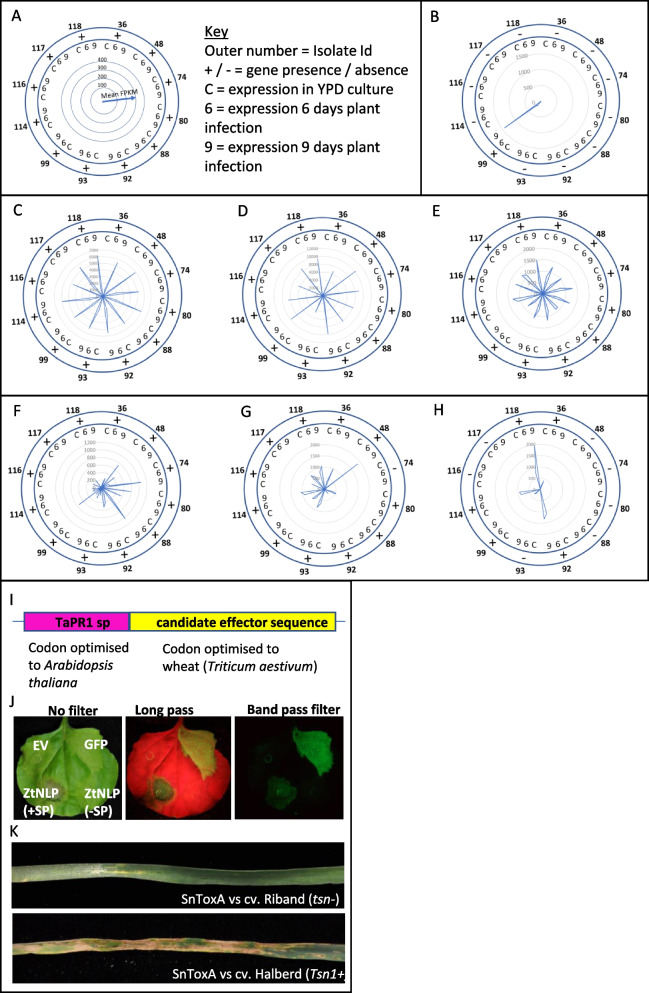
Expression variation of genes in the core and accessory pangenome and establishment of a transient viral overexpression system deployed for necrotrophic effector screens. **A** Key to interpreting data shown in the figures. **B** Presence/absence and gene expression variation for the *Succinate dehydrogenase subunit C* paralogue present in the accessory genome. **C**–**E** Expression profiles of the ZtLysM effectors, *3LysM*, *1LysM* and *xLysM*, all present in the core pangenome. **F** Expression profile of the avirulence effector *AvrStb6*. Lack of any expression in isolate 118 classifies the effector into the accessory pangenome. **G** and **H** Represent examples of additional candidate effectors in the accessory pangenome. The figure illustrates that accessory genes display substantial expression variation in addition to presence and absence variation. **I** Development and adaptation of a universal standard viral (*Foxtail Mosaic virus*-FoMV) mediated expression system used for comparative screening of different fungal effectors secreted by the wheat *PR1* gene signal peptide system. **J** Transient agro-expression in tobacco confirms the ability of the TaPR1 signal peptide, in the FoMV vector, to deliver a functional active, cell-death-inducing fungal effector, the *Zymoseptoria* necrosis and ethylene inducing-like protein (ZtNLP) into plants. **K** Functional validation of the expression system to deliver a wheat cell death inducing necrotrophic effector. The *Parastagonospora nodorum* necrotrophic effector ToxA, delivered by the viral vector system, induced specific cell death (leaf necrosis) only in wheat genotypes with the corresponding sensitivity gene *Tsn1+*

WolfPsort protein localization predictions were run on all the proteins encoded by the pangenome. We then calculated the relative percentage of proteins predicted to localise to each sub-cellular region as a feature of the total number of proteins for the core or accessory pangenome (Fig. 4). Several localization categories were enriched in the core genome including “cytoplasm”, “extracellular” and “plasma membrane”, the latter of which contains many key transport functions (Fig. 4). On the contrary, the locations “nucleus” and “mitochondria” were associated with a higher percentage of genes in the accessory genome (Fig. 4). The accessory genome was enriched for particular zinc-binding domains which may provide for transcription flexibility. The enrichment of this category was also reported in a previous pangenome study highlighting that our overall pipeline generated comparable results [27, 28]. The “mitochondrial” enrichment is interesting as there exist pseudogenes and paralogues of bona-fide mitochondrial proteins which are targeted by antifungal chemistries. One clear example in the accessory pangenome is a paralogue of a succinate dehydrogenase subunit C (SDHC3) which has been shown to mediate standing resistance towards a subclass of SDHI (succinate dehydrogenase inhibitor) fungicides in *Z. tritici* [45]. We identified complete sequences of this gene in only three of the seventeen sequenced isolates, and we detected a high level of gene expression in only one of the 12 subsequently studied by RNAseq (Fig. 5B). Nevertheless, the previous study, and our current analysis, emphasise that there are indeed important functions in the accessory genome but that these are probably more important for adaptation to changing environments, than for the core lifestyle.

### Effector proteins in the accessory genome display more inter-strain variability in expression than those in the core

Over eight percent of the genes in the core genome encoded putative secreted proteins, a number higher than that for the accessory genome (~5%). However, genes in the accessory genome encoding secreted protein were more frequently of unknown function, lacking recognisable domains or catalytic regions. These proteins of unknown function amounted to ~75% of the total accessory secretome relative to 42% for the core secretome. Many of the candidate (and in some cases validated) effectors present in the core genome were expressed in similar patterns, and to comparable levels, in all twelve isolates analysed by RNASeq. For example, the three LysM (Lysin) domain-containing effectors, *3LysM*, *1LysM* and *xLysM*, were expressed to very similar levels, and in identical patterns, by each strain (Fig. 5C–E). The 3LysM effector remains the only major secreted virulence determinant of *Z. tritici*, where this protein serves to supress chitin-triggered plant immunity during early infection [46, 47]. However, all three LysM effectors are known to work together to this end [48]. It is thus noteworthy that there was very little expression variation for these genes between isolates, re-enforcing suggestions that this defence suppression is a core component of infection. In contrast, candidate effectors found in the accessory genome displayed both presence and absence variation, and highly variable expression between isolates (Fig. 5F–H). For example, *AvrStb6*, the first avirulence effector identified in *Z. tritici*, [49, 50] displays considerable expression variation (Fig. 5F), despite being present in all isolates. This sequence variable, small, secreted protein, is recognised by wheat cultivars containing functional alleles of the receptor-like kinase *Stb6* [25, 51]. This recognition provides disease resistance against all isolates which harbour a particular allele of this effector. There was significant expression variation of *AvrStb6* between isolates and we identified one isolate (Zt118) where we failed to detect any expression at all, in any of the three conditions tested. For this reason, in our prediction pipeline, *AvrStb6* was classified into the accessory genome. Many other candidate effectors of currently unknown functions displayed significant presence/absence and/or expression variation in the accessory genome (Fig. 5G and H).

### Functional screens provide no evidence for necrotrophic effector activity in either the core or accessory pangenome

We adopted a recently developed viral vector-mediated protein transient expression system for wheat (and other cereal and non-cereal crops), utilising the *Foxtail Mosaic virus* (FoMV), to perform a medium- to high-throughput screen of candidate *Z. tritici* effectors from the pangenome. We specifically aimed to test whether any had the ability to induce necrosis on a small panel of wheat cultivars, which might imply their function at the transition to disease symptoms as “necrotrophic effectors” [36, 52]. For this study, we made some modifications to the previously published system [53]. To drive extracellular protein secretion in wheat, we used the wheat *pathogenesis-related protein 1* (*TaPR1*) signal peptide sequence, codon optimised to *Arabidopsis thaliana*, which was then fused in-frame with each candidate fungal effector sequence by gene synthesis (Fig. 5I). The efficacy of the TaPR1 signal peptide to secrete functional *Z. tritici* extracellular proteins was established by its ability to induce leaf necrosis in tobacco, when placed in front of the *Z. tritici* necrosis and ethylene inducing-like protein (ZtNLP), which is functional only when targeted for extracellular secretion (Fig. 5 Jl) [54]. We then confirmed that the vector could overexpress proteins equally well across a range of wheat cultivars by visualising local and systemic expression of the green fluorescent protein (GFP) throughout the leaves of different wheat cultivars (Additional file 2: Fig. S3). Finally, we then tested the ability of the well characterised necrotrophic effector SnToxA, from the related wheat pathogen *Parastagonospora nodorum*, to induce necrosis in wheat leaves of genotypes possessing the *Tsn1* sensitivity gene [55]. As anticipated, FoMV-mediated overexpression of SnToxA, with the TaPR1 signal peptide, induced necrosis only on the wheat cultivar possessing *Tsn1* (cv Halberd in Fig. 5K) with no symptoms observed on cultivars lacking this susceptibility gene (cv Riband in Fig. 5K). These data confirmed that the viral vector-mediated overexpression system was suitable for a larger scale screening of candidate *Z. tritici* effectors to identify any with necrotrophic effector activity. A total of 88 candidates were selected and screened against five wheat cultivars (Additional file 5: Table S3). These encompassed 66 proteins residing in the core pangenome and 22 from the accessory. The genes selected (Additional file 5: Table S3) ranged from core genes with no strain-specific polymorphisms (monomorphic) and high in planta expression, through to those in the accessory genome exhibiting presence/absence and/or substantial expression polymorphism. In all experiments, parallel ToxA vs cv Halberd and cv Riband technical controls were run, and these always produced the anticipated results. In contrast, none of the 88 tested *Z. tritici* overexpressed proteins induced any leaf necrosis on any wheat cultivar. In summary, these results did not provide any evidence for necrotrophic effectors, and therefore, no support for any of the tested candidates in inducing the switch to necrotrophic growth during infection.

### Core genes encoding proteins with demonstrated, or predicted, essential-for-life or key virulence functions have lower overall levels of amino acid polymorphism

**Fig. 6:**
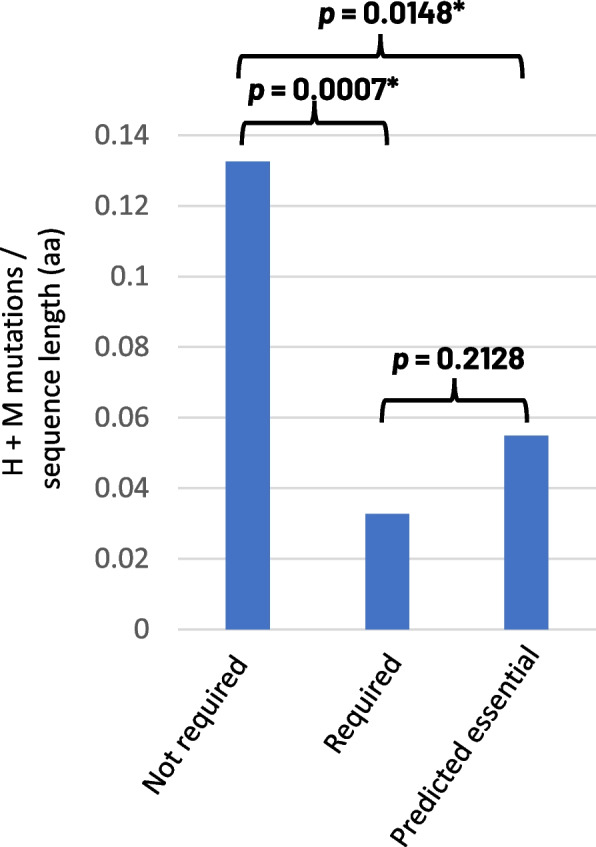
Analysis of core gene sets of experimentally validated pathogenicity and/or predicted essential-for-life genes reveals lower mutation rates than seen in non-essential genes. The average high and moderate (H/M) mutation rates expressed as a feature of protein lengths (aa) for gene lists encoding proteins which have been; 1—experimentally determined to play no (or very minor) roles in fungal virulence; 2—experimentally determined to play an important role in virulence and; 3—predicted to encode putative essential-for-life genes through orthology to proteins experimentally characterised in *Aspergillus fumigatus.* Asterisk (*) symbol indicates a statistically significant difference between mutation rates between the indicated gene sets. Gene lists and associated polymorphism data is shown in Additional file 6: Table S4

We performed a literature search to identify all *Z. tritici* genes which had been subject to functional characterisation, either in virulence, or as essential genes dating up to 2018. This generated a list of 28 protein sequences (Additional file 6: Table S4). Similarly, we identified a list of 26 proteins which had also been functionally characterised but had been shown to not play a major role in virulence or be essential (Additional file 6: Table S4). We then investigated the levels of cumulative redundant high and moderate mutations in these genes and expressed their values as a feature of each protein length and then determined the average values for the set. Figure 6 highlights that the published genes affecting virulence had a statistically significant lower frequency (*p =* 0.007) of mutation (polymorphism) than those which have been shown to be functionally redundant.

Studies aimed at defining essential-for-life (essential) genes in filamentous fungi (excluding yeasts) are few, and to date only one representative exists in the database of essential genes (DEG), that being from the opportunistic human pathogen *Aspergillus fumigatus* [56–58]. We identified the *Z. tritici* orthologue of all 28 genes experimentally shown to be essential for life in *A. fumigatus* and again calculated the relative mutation rates of each relative to sequence lengths (Additional file 7 Table S5). Once again, we found a clear statistically significant reduction (*p =* 0.0148) in the frequency of high and moderate SNP mutations in these candidate essential genes, than observed for genes shown to be not essential for either virulence or life (Fig. 6). In contrast, there was no significant difference (*p =* 0.2128) between mutation rates for experimentally validated virulence genes and the predicted essential genes (Fig. 6). These data together support the concept that levels of amino acid polymorphisms in populations could be used to predict the relative importance of genes for the core lifestyle of the organism.

### The utility of the combined predictive approach to reveal pathogen weakness is supported through genetic complementation studies on a five “core” gene deletion mutant

**Fig. 7:**
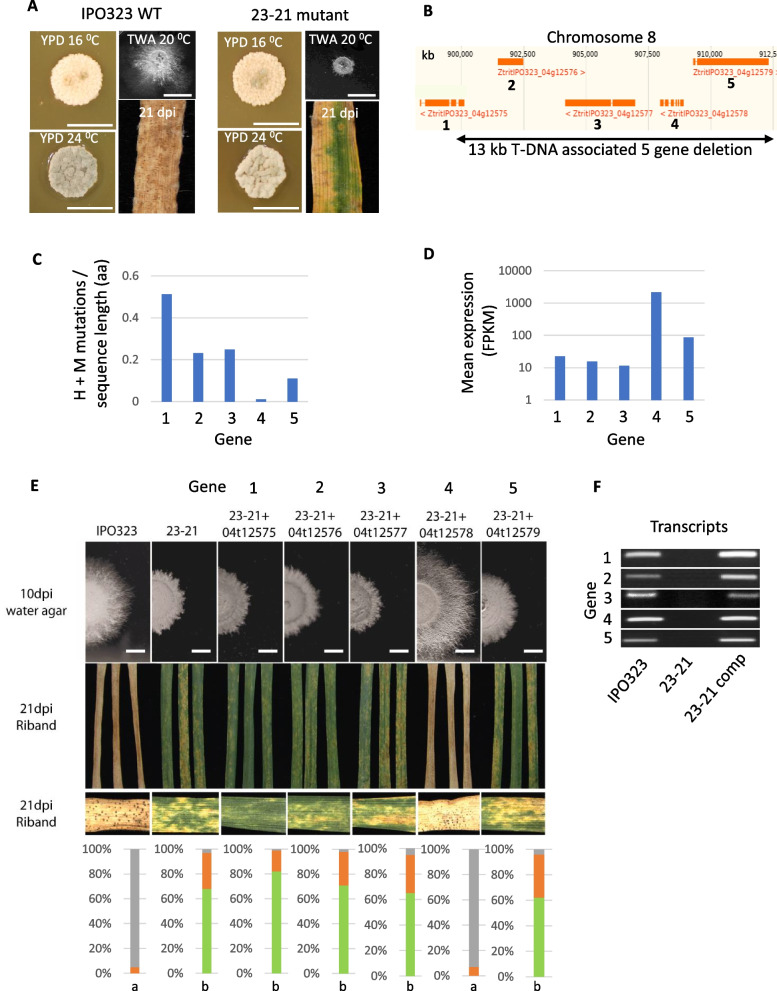
Functional complementation assays on a five-gene deletion non-pathogenic *Z. tritici* mutant support the combined use of pangenome-derived mutation rate and expression level analysis as predictors for important core lifestyle genes. **A** Growth and infection characteristics of a *Z. tritici* random T-DNA insertion mutant “23-21”. The strain grows normally on rich nutrient agar but is defective in filamentous growth on poor nutrient agar and severely compromised in wheat leaf disease causing activity. Scale bars represent 1 cm. **B** Whole genome resequencing of strain 23-21 reveals a T-DNA mediated deletion of a 13 kb genomic region disrupting or deleting five predicted genes from the core pangenome. **C** Displays the average High and Moderate mutation events for each gene (labelled 1-5) from the pangenome relative to encoded protein length (aa). **D** Displays the mean relative expression levels of genes 1-5 across 12 strain-specific RNAseq datasets. **E** Functional complementation assays. Strain 23-21 had each of the five genes re-introduced separately by genetic complementation. Upper panels show effects on filamentous growth on poor nutrient agar. Middle panels show representative plant infection images at 21 dpi for one of multiple tested complemented isolates for each gene. Lower panel shows the quantification of disease levels using Lemna Grid image analysis system. Complementation by gene 4 encoding a predicted nucleoside diphosphate kinase (NDK) alone resulted in full restoration of pathogenicity in the 23-21 mutant. “a” and “b” indicate statistically significant differences (*p* < 0.001) between green leaf areas for the tested treatments. **F** RT-PCR analysis of each complemented strain, demonstrating that all constructs were correctly expressed by their native promoters in all tested isolates

*Z. tritici* is amenable to forward genetics screens based on random integration of T-DNA via *Agrobacterium*-mediated fungal transformation (AMT). Our previous work had generated one *Z. tritici* T-DNA mutant in particular which was unable to cause full disease on infected wheat leaves (Fig. 7A). This mutant, called 23-21 (the 23rd round of transformation and the 21st colony picked), could grow normally as wild type on nutrient-rich agar medium (YPD) but failed to extend comparable levels of fungal hyphae when grown on nutrient deprived medium, including water agar (Fig. 7A). We used whole-genome Illumina-based re-sequencing of 23-21 to reveal the position of the potentially causative T-DNA insertion. This analysis revealed a single T-DNA integration between positions 899878 and 912699 on chromosome 8, causing a deletion/disruption of five predicted coding sequences (Fig. 7B). SNP and indel analysis revealed no additional un-tagged mutations in the 23-21 strain relative to the wild type genome sequence of IPO323.

The 23-21 T-DNA deletion effectively removed five genes, all of which reside in the core genome, and with the following functional (Interpro) predictions; gene 1 = *Cytochrome P450*; gene 2 = *S-adenosyl methionine methyltransferase*; gene 3 = *Zinc (2) C6 type DNA binding protein*; gene 4 = *Nucleoside diphosphate kinase (NDK)* and gene 5 = *Glycosyl hydrolase 31* (Fig. 7B). We investigated the relative numbers of high and moderate mutations in each protein as a feature of protein length, which revealed that protein 4 (NDK) had far fewer mutations affecting amino acid sequence changes than the other four analysed sequences across the isolate set (Fig. 7C and Additional file 2: Fig. S4). We then investigated the average relative expression of all five genes across all the tested isolates. This also revealed that gene 4 (encoding the NDK) was significantly higher expressed than all of the other candidates (Fig. 7D and Additional file 2: Fig. S5). We thus hypothesised that the loss of the least-polymorphic and highest-expressed gene, gene 4, the NDK, was responsible for the loss of virulence, and associated defect in extending hyphal filaments. To test this, we transformed each of the five candidate genes back individually into the 23-21 *Z. tritici* mutant strain, each driven by their own endogenous promoter. Multiple transformants were obtained from each and retested for virulence on wheat leaves and ability to extend filamentous hyphae on water agar. This demonstrated that re-introduction of the *NDK* gene alone (gene 4) fully restored both virulence and hyphal growth to wild type levels (Fig. 7E and Additional file 2: Fig. S6). Reintroduction of each of the other four candidates did not cause any change in phenotype from the original mutant strain 23-21 (Fig. 7E). To ensure that all re-introduced genes were expressed in their respective complemented strain, RT-PCR was done on each of the transformants and the original 23-21 mutant. This confirmed the anticipated lack of each transcript in the 23-21 mutant and demonstrated correct expression of each target gene in the complemented isolates (Fig. 7F). Thus, re-introduction of the single gene with least population-level polymorphisms of the five candidates, and highest relative expression, restored the defective phenotypes in this wheat pathogen. This experimental observation supports the utility of combining pangenomics and transcriptomics for predicting genes which are potentially important for key functional life traits.

## Discussion

### Combined pangenomics and transcriptomics can be used as weapons against rapidly evolving pathogens

Pangenomics analyses have been performed on other fungi and yeasts including *Saccharomyces cerevisiae*, *Candida albicans*, *Cryptococcus neoformans* var. *grubii* and *Aspergillus fumigatus* [10]*.* All four species are model organisms in eukaryotic genomics and the latter three can also cause human diseases. Recent pangenome analyses on each of these species revealed that > 80% of all genes detected were core and thus found in every strain [10]. Our current study, and those previously [27, 28], clearly highlight that *Z. tritici* has a larger (> 40% of the total genes) accessory genome than these species. Why and how *Z. tritici* and potentially other plant pathogenic fungi maintain such a large accessory component is unclear. For example, the smallest eight chromosomes identified in IPO323 do not have any clear major impact in virulence, or any other processes, with only one study currently suggesting chromosomes 14, 16, 18, 19 and 21 as playing a subtle role in virulence on selected wheat cultivars [59]. Nevertheless, combinations of these small chromosomes are maintained in populations. It has been suggested that they provide for recombination events which may underlie the ability of the fungus to evolve rapidly to adapt to environmental stresses, potentially combined with high rates of activity of transposable elements [60–62]. A similar case may be argued for the large accessory genome. Support for this is provided by the functional importance of two accessory genome components referred to in this study, the SDH subunit C paralogue and AvrStb6, both of which have key roles in interacting with variable elements of the external environment (fungicide application and cultivars with matching disease resistance genes, respectively) [25, 45]. Other biological processes have been described which can also be lost in individual *Z. tritici* isolates without clear fitness defects including control of melanisation (pigmentation) [63].

Our study has highlighted that the accessory, rapidly evolving genome of *Z. tritici* could potentially be used against it, when the focus is instead placed on what cannot be lost or has limited polymorphism. This study has sought to do this, by considering core genes as not only present and functional in all isolates but also expressed by all isolates. We also took the approach of attempting to rank gene sets in the core genome, based on levels of amino acid sequence variability. There are of course some caveats with the methods we have used, particularly in that redundant (identical type and position) mutations cannot be easily separated from a similar number of mutations which may occur at unique positions in each isolate (non-redundant mutations). Thus, our method is not suited to rank rapidly evolving genes. Nevertheless, the method enables genes with no, and low, variability to be easily identified based on low rates of polymorphisms affecting amino acid changes. The approach clearly demonstrated that experimentally verified and predicted essential-for-life genes or virulence genes harboured significantly less polymorphism of this type between isolates, than those whose functions have been shown to be dispensable for these processes. Finally, we extended this approach to identify a new virulence function of a nucleoside diphosphate kinase (NDK), which exhibited the least variation of all genes in a 5-gene T-DNA deletion. Based upon these findings, we propose that levels of polymorphism in core genes identified in pangenomics could be used to infer important functions for the encoded proteins and thereby prioritise genes for functional analysis to identify new targets for crop protection and potentially animal health.

### An emerging weakness in the infection biology of *Z. tritici* and related pathogens?

This study also provided further specific insights into the infection biology of this important wheat pathogen. Firstly, no evidence was obtained supporting the presence of major necrotrophic effector activities for 88 secreted proteins selected from both the core and accessory genomes. We cannot discard the possibility that we did not select the correct proteins or that multiple effectors could work together to invoke plant cell death (also suggested by similar expression profiles). If the latter is the case, this would differ from the clear importance of single necrotrophic effectors in related wheat-infecting fungi, notably *Parastagonospora nodorum* and *Pyrenophora* species [35]. It is also possible that the expression levels we derive from the transient FoMV system are lower than those required for effectors whose recognition is less pronounced than that of SnToxA. Nevertheless, the data presented herein provides no support for the idea that the transition to symptomatic growth, and plant cell death is invoked by the recognition of major (single) necrotrophic *Z. tritici* effectors. It is therefore possible that the mass production of these proteins at the onset of symptoms may instead serve to protect fungal hyphae from the apparent hyperactivation of cell death and plant response. Consistent with this, secreted effectors from this and other fungi have been shown to have the ability to inhibit plant-derived proteases, chitinases and other cell wall attacking enzymes which are themselves induced by plants during defence responses [46, 48, 64, 65]. More recently, fungal effectors have also been shown to manipulate and remodel micro- and mycobiomes [66, 67].

The identification of a key role in virulence for NDK is however significant. These proteins have been ascribed multiple functions from different prokaryotic and eukaryotic organisms. In fungi, there is one report ascribing a role for modulation of plant immunity [68], whilst in another species (*A. fumigatus*), an essential-for-life function has been demonstrated [69]. However, the primary and conserved function for these proteins is to phosphorylate nucleoside diphosphates, usually using ATP as substrate, to generate other nucleoside triphosphates (NTPs) required to help fuel various cellular processes [70, 71]. This is particularly important when limited resources make generation of certain NTPs by other means more difficult. Thus, NDKs present a key salvage pathway activity for regeneration of NTPs most likely important when extracellular resources are limited. Important roles for a number of other salvage pathways, and in particular biosynthetic pathways, have recently emerged from other gene function studies on *Z. tritici*. For example, lysine biosynthesis has been shown to be essential for infection and hyphal growth on poor nutrients [72], as has purine biosynthesis [73]. These functions are redundant for growth on complete media. Previous transcriptome data on the very early stage of leaf surface colonisation has clearly indicated that the fungus is in a nutrient-depleted environment and is reliant on utilising intracellular stores [33]. Taken together with the previous functional studies, and the key role now shown also for NDK, it is clear that *Z. tritici* is extremely vulnerable to inhibition of key biosynthetic and salvage pathways when on the leaf surface during early colonisation, and potentially through to the induction of plant cell death some 7–10 days later. Thus, new crop protection products or strategies could be directed towards the inhibition of these processes, which may represent a weakness in *Z. tritici*, and potentially for a wide variety of other related fungi with similar modes of infection.

## Conclusions

This study provides initial support the combined use of pangenomics and transcriptomics for defining genes which represent core, and potentially exploitable, weaknesses in rapidly evolving pathogens. However, in principle, the approaches could be used for gene prioritisation in any biological system where multiple genomes and transcriptomes are available. We anticipate that these approaches could advance discovery pipelines of core biological processes in many diverse biological systems.

## Methods

### Virulence assay on a European collection of *Z. tritici* isolates

A collection of *Z. tritici* strains, 42 in total, were recovered from wheat fields spanning 11 European countries during 2015–2016. All isolates, including one of the community reference isolates, IPO323, were subsequently maintained as 50% glycerol stocks at – 80 **°**C prior to use. The plant material included 21 cultivars and landraces of hexaploid wheat (*Triticum aestivum* L.). These genotypes were selected based on their universal disease susceptibility in various field trials across several years and locations (data not shown).

*Z. tritici* inoculation of wheat was performed in an environment-controlled glasshouse facility (17 **°**C day/night, 60% relative humidity, 16/8 h light/dark) as previously described [47]. The spore concentrations used for all strains was 1 × 10^6^ per ml in sterile water containing 0.1% Tween20. Chlorosis-and-necrosis-covered leaf area (CNCLA) was visually scored at 21 days post inoculation (dpi) on a scale of 1 to 5 representing 0–100% coverage with 20% confidence interval. The infected leaves were also photographed with a Nikon D90 camera for subsequent automated scoring using the LemnaGrid (LemnaTec, Aachen, Germany) image analysis software. The image analysis pipeline is summarised in Additional file 2: Fig. S1. In brief, photographs were pre-processed using the Windows 10 Photos software to trim the images to the assay area and to manually separate any overlapping leaves with drawn lines. The images were then imported into the LemnaGrid software. Leaves were segmented based on a combination of intensity thresholding and a colour-based classification. Ultimately, the proportion of diseased tissue for each leaf was quantified using a second, customised, colour-based classification (3 classes: chlorotic, necrotic or healthy). Scores were then exported for subsequent quantitative and statistical analysis.

For quantification of asexual spores, following photography, the inoculated part of each leaf was harvested and incubated in a 15-ml falcon tube with a wet cotton wool at 15 °C for 24 h to induce sporulation and extrusion of spore masses from leaf stomata. Spores were then washed from the leaves by adding 2 ml of sterile distilled H_2_O with 0.05% Tween 20 to each falcon tube followed by a 1 min vortex. Spore quantification was carried out by using a spectrophotometer with a standard curve generated from a serial dilution of *Z. tritici* spores. Data were visualised by Heatmap2 (Galaxy Version 3.0.1) with Euclidean distance method and complete clustering method in Galaxy (https://usegalaxy.org/). Each cultivar vs isolate assessment was performed twice (in two replications), with three 5-cm-long leaf segments inoculated and analysed per replication resulting in a total of 6 independent seedling leaves tested by each isolate.

### *Z. tritici* genome sequencing, assembly and quality control

Fungal materials (blastospores) were generated by culturing *Z. tritici* isolates in Yeast Potato Dextrose (YPD) broth at 80 rpm and 15 °C for 5 days. Genomic DNA was extracted by using the Illustra Nucleon Phytopure kit (GE Healthcare) and purified by the DNeasy Plant Mini Kit (Qiagen) according to the manufacturers’ protocols respectively and subsequently sent for genome sequencing by Illumina HiSeq2500 with a 250 bp paired end read metric and a target of 100× coverage (Earlham Institute, Norwich, UK). Quality control of sequencing reads was performed using Fastqc (v0.11.9, https://www.bioinformatics.babraham.ac.uk/projects/fastqc/). Trimmomatic (v0.38-Java-1.8, http://www.usadellab.org/cms/?page=trimmomatic) was used to eliminate any remaining Illumina adaptors [74]. Genomes were assembled by Discovardenovo (v52485, https://software.broadinstitute.org/software/discovar/blog/). Decypher BLAST and Megan were used to check for any sequence contamination. Genome completeness was assessed by BUSCO 3.0 against the pezizomycotina_odb9 database [75, 76].

### *Z. tritici* pangenome construction, annotation and in silico characterisation

In total, 17 *Z. tritici* genomes were generated by the sequencing project in this study. To construct a pangenome, novel region discovery was conducted by Panseq (https://lfz.corefacility.ca/panseq/page/novel.html) with IPO323 as a reference and 17 *Z. tritici* isolates as queries with 85% identity cut-off [77]. Default settings were applied (minimum novel region size: 500, nucmer values (b: 200, c: 50, d: 0.12, g: 100, l: 20)). The annotation was produced using the RRes IPO323 annotation (R. King, Zym_tritici_RRes_v4.0_RK_public.gff (2017)p. , doi:10.6084/m9.figshare.4753708.v1 [78]) and then annotating novel sequence in relation to the IPO323 reference using the Maker2 pipeline using AUGUSTUS with a custom *Z. tritici* species model matrix and self-trained GeneMark (version 4) with RepeatMasker (v4.50, http://www.repeatmasker.org) [78–80]. A transcriptome assembly using RNAseq from 12 out of these 17 isolates (see below) was supplied as evidence to Maker2.

Blast2GO v.3.1 was used with BLASTP searches with an *E*-value of 0.001 against the NCBI nr database from 15/01/18, filtered using blast2go annotation algorithm with settings, *E*-value filter 0.000001, annotation CutOff 55, GO weight 5, Hsp-Hit Coverage CutOff 0 and GO and enzyme code annotated using a local GO database from 07/2017. Interproscan (v66.0) results were imported into Blast2Go and the GO annotations merged. The pangenome fasta file and its annotations (gtf) are provided in Additional file 8: Data S2 and Additional file 9: Data S3 respectively.

Signal peptide and transmembrane domain proteins were extracted from Blast2Go. ProtComp (Version 9.0) (Softberry, USA) was used to exclude GPI anchored membrane proteins and other non-extracellular loci proteins. WoLfPSort was used to identify final destination and big-PI to further remove GPI-anchored proteins. Putative effector proteins were predicted using EffectorP (v2.0).

To identify core and accessory genes, five bases were removed from the 5′ end of the reads, and a sliding window crop (size four bases, quality Q20) was used to shorten reads. Reads smaller than 40 bases after trimming were removed. This removed < 1% of raw reads. Reads were mapped to the PanSeq pangenome reference using BWA (v0.7.17-intel-2018a, http://bio-bwa.sourceforge.net/) [81]. BedTools (v2.28, https://bedtools.readthedocs.io/en/latest/) was used to generate mapped gene counts and lengths. Count data for each gene was imported into an SQLite database (v3.25.2, https://sqlite.org/index.html). Gene Coverage was calculated for each gene using the counts, and the read and gene lengths using the formula *C* = *LN*/*G*, where *C* is the coverage, *L* is the read length, *N* is the number of reads and *G* is the gene length. The coverage of all IPO323 genes for each accession was used to generate a mean coverage for each accession, and this was used to generate a scaled accession coverage for all genes. Genes were flagged absent if their scaled coverage was less than 35% of the accession coverage mean.

### Phylogenetic analysis on isolates

Six sequences were used for phylogenetic strain analysis, including 18S (1799bp, full length), EF1alpha (1-1309bp trimmed from potential full length of 1389), ITS1 (141bp), ITS2 (161bp), RPB1 (RNA polymerase II largest subunit 5322bp, full length) and RPB2 (RNA polymerase II second-largest subunit 3759bp, full length). All sequences were identified from all 17 Zt genomes using the IPO323 sequences as Blastn queries (Additional file 3: Data S1). The ITS1 and ITS2 sequences from NCBI Reference Sequence: NR_158992.1). All ITS1 and ITS2 sequences extracted from all isolates were 100% identical. Full length sequences were analysed for all except EF1alpha, because the last 80 bp were sequenced poorly and could not be assembled. Sequence alignment was done for concatenated sequences following this order 18S-EF1alpha-ITS1-ITS2-RPB1-RPB2. SplitsTree4 (version 4.18.3) was used to construct the networks using the concatenated sequences of 6 house-keeping genes from the 18 isolates. Sequences were aligned in Geneious with Clustal Omega and exported to SplitsTree4 which was run on Default settings.

### Single nucleotide polymorphism (SNP) analysis of genes predicted for the pangenome

SNP analysis for all genes present in each sequenced *Z. tritici* isolate was done in Galaxy. Reads of each isolate were mapped against the constructed pangenome by Bowtie2 (Galaxy version 2.3.4.3) [82]. Aligned records in BAM datasets were examined by MarkDuplicatesWithMateCigar (Galaxy version 2.7.1.0) to locate duplicate molecules. Positional distribution of insertions and deletions in the BAM datasets were re-aligned by BamLeftAlign (Galaxy version 1.0.2.29-1), and indels were identified. Bayesian genetic variants were detected by FreeBayes (Galaxy version 1.0.2.29-3) and filtered by SNPSift Filter (v4.3T). SNPs kept were homozygous, with a quality equal or above 30, and a read coverage between 25 and 300% the average coverage of each isolate specific genome coverage [83]. These filtering conditions were based upon removing false positive SNPs using re-sequencing of IPO323 as a control against the IPO323 reference. Filtered variants were annotated by SNPEff (v4.3T) [84]. Variants with a value of (SAF-SAR)/(SAF+SAR) < − 0.5 or (SAF-SAR)/(SAF+SAR) > 0.5 were removed (SAF, number of alternate observations on the forward strand; SAR, number of alternate observations on the reverse strand). Variants of the following predicted effects were also removed, which include 3 or 5 prime UTR variant, intergenic region, intragenic variant, intron variant and non-coding transcript variant. Partially redundant cumulative numbers of moderate and high effect SNPs in each predicted protein were generated in Excel and subsequently expressed as a frequency value relative to protein length. All predicted high effect mutations resulting from frame shifts were removed from the analysis as many appeared in tandem as complementary compensatory (corrective) pairs [85], which together did not result in protein inactivation. We subsequently calculated for each gene, the total cumulative redundant mutations (high, moderate and modifier), observed across the 17 isolates, compared to the IPO323 reference and divided it by the length of the encoded protein. We also did the same for the grouped High and Moderate mutations and finally for the high effect mutations alone. This generated a numerical value which can be used to intercompare and/or rank the relative “variability” (or mutation rates) of genes and their encoded proteins (Additional file 4: Table S2).

### RNAseq analysis of *Z. tritici*

Based on the high virulence levels and distinct virulence profiles in the initial wheat infection screen, 12 out of 17 *Z. tritici* isolates, whose genomes were sequenced, were selected for transcriptomic analysis. A single susceptible wheat cultivar “Panorama” (Limagrain Ltd) on which most isolates displayed similar kinetics of infection was used for sample preparation as described previously [32]. Samples were harvested at 6dpi (within the symptomless phase) and 9dpi (transition phase spanning appearance of first visible disease symptoms). Each sample consisted of 3 independent pooled seedling leaves, and three biological replicate samples were generated for each condition/time point for each isolate. Each isolate was also grown and harvested during log phase growth in YPD broth to investigate gene expression away from the wheat plant. Again, this was performed in triplicate. Total RNA was extracted by using TRIzol (Invitrogen) and subjected to transcriptomics analysis by Illumina Hiseq4000 with a 100 bp paired-end read metric (BGI genomics, Beijing, China). Both in vitro and in planta samples were mapped to the pangenome using Hisat2 (v2.0.5). Mapped reads were counted using featureCounts (v1.6.2).

### In planta necrotrophic effector screen using *foxtail mosaic virus*-mediated overexpression (FoMV VOX)

Gene expression and sequence variation of genes together with extracellular secretion prediction from all, or select members of the 12 *Z. tritici* isolates, was used for effector candidate selection. *Foxtail mosaic virus* was used to overexpress effector candidates in five distinct wheat cultivars, namely CV6_Gabrio, CV7_KWS Kielder, CV12_Pakito, CV13_Pamier, CV14_Panorama. In total, 88 effector candidates were selected from *Z. tritici* based on different criteria including protein length, cysteine content, in planta expression profile and sequence polymorphism. The most frequent allele or allele from the most virulent isolates were chosen for overexpression analysis. In order to improve in planta overexpression of effector candidates, the secretion signal sequences of selected effector candidates were replaced with a universal wheat PR1 secretion signal sequence (GenBank: HQ848391.1) which was codon-optimised for *Arabidopsis thaliana* to avoid RNA silencing of wheat *PR1* gene caused by FoMV. Mature effector candidate sequences were also codon-optimised for wheat. Modified effector candidate genes were synthesised (ThermoFisher Scientific) and cloned into the target vector pGR-FoMV.PV139.sgp101.MCS (FOMV) by using double restriction enzyme cloning (ClaI and XbaI). Recombinant vectors were transformed into *Agrobacterium tumefaciens* GV3101 through electroporation. Plant growth conditions and inoculation of *Nicotiana benthamiana* and *T. aestivum* with effector-candidate-expressing constructs were performed according to previous published study [53]. Both the local and systemically infected leaves were observed at 7 and 14 dpi for necrosis or chlorosis induction and at 21 dpi for systemic viral symptoms. Downstream processing of datasets generated through photography, stereomicroscopy and ELISA detection of FoMV coat protein were performed as described in previous studies [53]. Each viral vector construct containing candidate effectors was inoculated onto 5 independent wheat seedlings, and each overall experiment was performed twice (resulting in 10 leaves screened/wheat genotype).

### Molecular characterisation of the *Z. tritici* IPO323 mutant strain “23-21”

The generation of a collection of *Agrobacterium*-mediated random mutants of isolate IPO323 was described previously [78, 86]. Strain 23-21 emerged from this screen. Genomic DNA was extracted from this strain (Illustra Nucleon Phytopure) and sent for genome re-sequencing (BGI genomics), and the position of the T-DNA insertion was then determined. Paired end reads were mapped to the plasmid reference, and where one mate was mapped and the other mate was unmapped, this unmapped read was likely to be from the boundary of the plasmid insertion site. Therefore, these unmapped reads were extracted and mapped back to the fungal genome sequence. A stack of forward reads represents the boundary of the 5′ plasmid insertion boundary loci and the stack of reverse reads represents the 3′ plasmid insertion boundary loci. The gap between these stacks represents any deleted sequence. Copies of plasmid insertion are determined by the average coverage of mapped reads to the plasmid versus mapping the raw data to the fungal genome. Detailed instructions on running the FindInsertSeq workflow are available in a previous publication [87]. The datasets returned from these analyses revealed the deletion of a region of ~13kb generating loss of function for five genes.

To determine which gene(s) might be responsible for the loss of virulence we undertook a genetic complementation approach. In summary, each genomic sequence and its upstream and downstream region was amplified from WT genomic DNA and cloned (Gibson Assembly, NEB) into vector pCGEN [86]. *Agrobacterium*-mediated fungal transformation was done to re-introduce each missing gene individually into the 23-21 mutant via previously described protocols [54, 88].

Positive transformants were validated by PCR using genomic DNA as templates. At least five transformants were analysed for each complemented strain. Total RNA was extracted for positive transformants and restored transcript expression was tested by RT-PCR using gene specific primers (Additional file 10: Table S6). Three validated expressing isolates for each re-introduced gene were analysed for growth on 1% Tap Water Agar, to investigate ability to generate hyphal filaments, and subsequently tested for virulence on susceptible wheat cultivar Riband as per established assays [33, 78, 86]. Phenotype of growth on water agar and on plants were photographed at 10dpi and 21dpi, respectively. Infection assays were performed twice with a total of 6 independent seedling leaves scored for each tested strain.

## Supplementary Information

**Additional file 1: Table S1.** Details of the *Z. tritici* isolates analysed in the primary virulence screen.

**Additional file 2: Figure S1.** LemnaTec, LemnaGrid quantitative analysis of wheat leaves infected by *Z. tritici.* **Figure S2.** Gene presence/ absence polymorphism across the remaining chromosomes of reference strain IPO323. **Figure S3.** Wheat cultivar testing of FoMV driven GFP expression. **Figure S4.** The distribution of data points relating to the combined High (H) and Moderate (M) SNP mutations detected across 17 *Z. tritici* strains relative to IPO323. **Figure S5.** The distribution of gene expression data for genes 1-5 affected by T-DNA deletion in non-pathogenic isolate 23-21. **Figure S6.** The distribution of leaf infection data for WT, 23-21 mutant and gene 1-5 complementation strains.

**Additional file 3: Data S1.** The reference sequences used for phylogenetic analysis of *Z. tritici* isolates.

**Additional file 4: Table S2.** Complete information on all genes, their expression and characteristics of the encoded proteins for the pangenome.

**Additional file 5: Table S3.** List of the candidate effectors, and their predicted features, tested by FoMV-mediated overexpression in wheat.

**Additional file 6: Table S4.** List of *Zymoseptoria* genes functionally demonstrated to be either required or dispensable for pathogenicity and virulence on wheat. List contains genes characterised up to and including 2018.

**Additional file 7 Table S5.** *Zymoseptoria* orthologues of validated essential genes from *Aspergillus fumigatus.*

**Additional file 8: Data S2.** Complete genomic sequence for the pangenome.

**Additional file 9: Data S3.** Annotation file for the pangenome.

**Additional file 10: Table S6.** Oligos used in this study for complementation of strain 23-21 and for RT-PCR on the five affected genes.

**Additional file 11: Data S4.** Original gel for RT-PCR analysis on gene 1 complementation strains in 23-21. Note all oligos used span introns.

**Additional file 12: Data S5.** Original gel for RT-PCR analysis on gene 2 complementation strains in 23-21. Note all oligos used span introns.

**Additional file 13: Data S6.** Original gel for RT-PCR analysis on gene 3 complementation strains in 23-21. Note all oligos used span introns.

**Additional file 14: Data S7.** Original gel for RT-PCR analysis on gene 4 and 5 complementation strains in 23-21. Note all oligos used span introns.

**Additional file 15: Data S8.** Full Inventory of all data linked to this project.

## Data Availability

All strains and materials are available from the corresponding author. Data generated or analysed during this study are included in this published article and its supplementary information files which includes the PanSeq pangene sequences (Pangenome_Zt_17I_IPO323_v3.fasta) and annotations (annotation_pangenomveV3_2.gtf). All supplementary files are also freely available following publication from the Rothamsted Research repository (10.23637/rothamsted.98q90) [89]. The remaining raw project data (DNA and RNAseq read data and genome assemblies) is free for download on publication from NCBI and NCBI GEO using project code PRJNA890236 (https://www.ncbi.nlm.nih.gov/search/all/?term=PRJNA890236) [90]. Both raw and these derived expression files Accessible from NCBI GEO accession GSE222164 (https://www.ncbi.nlm.nih.gov/geo/query/acc.cgi?acc=GSE222164) [91].
