# Supplementary material for: Combined pangenomics and transcriptomics reveals core and redundant virulence processes in a rapidly evolving fungal plant pathogen
Source: BMC Biol. 2023 Feb 6;21:24. doi: 10.1186/s12915-023-01520-6 (PMC9903594; doi:10.1186/s12915-023-01520-6)
Supplement: Supplementary file 2 — Additional file 2: Figure S1. LemnaTec, LemnaGrid quantitative analysis of wheat leaves infected by Z. tritici. Figure S2. Gene presence/ absence polymorphism across the remaining chromosomes of reference strain IPO323. Figure S3. Wheat cultivar testing of FoMV driven GFP expression. Figure S4. The distribution of data points relating to the combined High (H) and Moderate (M) SNP mutations detected across 17 Z. tritici strains relative to IPO323. Figure S5. The distribution of gene expression data for genes 1-5 affected by T-DNA deletion in non-pathogenic isolate 23-21. Figure S6. The distribution of leaf infection data for WT, 23-21 mutant and gene 1-5 complementation strains. [file 12915_2023_1520_MOESM2_ESM.pptx]

## Slide 1
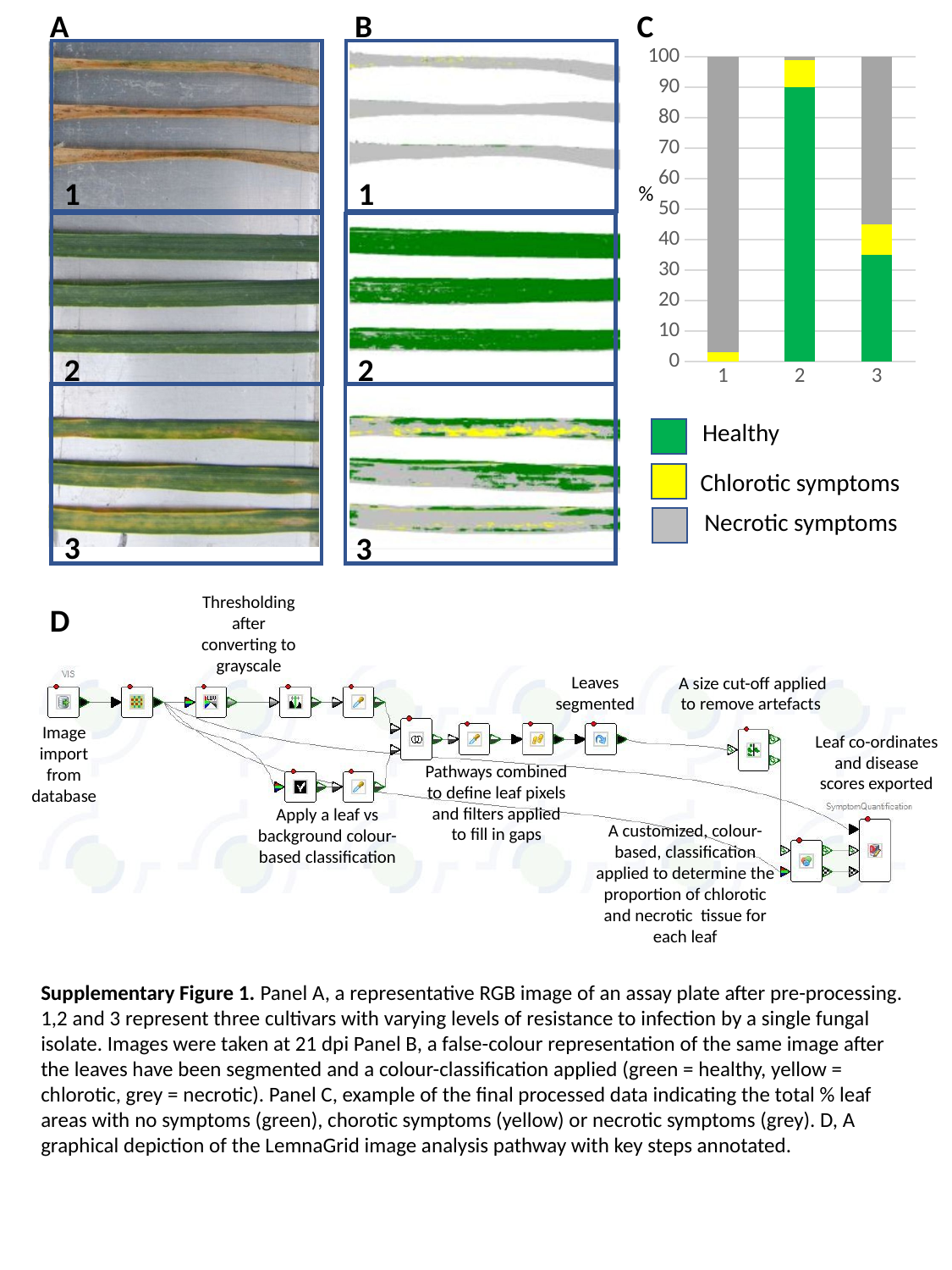

A
B
C
### Chart
| Category | | | |
|---|---|---|---|
1
1
%
2
2
Healthy
Chlorotic symptoms
Necrotic symptoms
3
3
Thresholding after converting to grayscale
D
Leaves segmented
A size cut-off applied to remove artefacts
Image import from database
Leaf co-ordinates and disease scores exported
Pathways combined to define leaf pixels and filters applied to fill in gaps
Apply a leaf vs background colour-based classification
A customized, colour-based, classification applied to determine the proportion of chlorotic and necrotic tissue for each leaf
Supplementary Figure 1. Panel A, a representative RGB image of an assay plate after pre-processing. 1,2 and 3 represent three cultivars with varying levels of resistance to infection by a single fungal isolate. Images were taken at 21 dpi Panel B, a false-colour representation of the same image after the leaves have been segmented and a colour-classification applied (green = healthy, yellow = chlorotic, grey = necrotic). Panel C, example of the final processed data indicating the total % leaf areas with no symptoms (green), chorotic symptoms (yellow) or necrotic symptoms (grey). D, A graphical depiction of the LemnaGrid image analysis pathway with key steps annotated.

## Slide 2
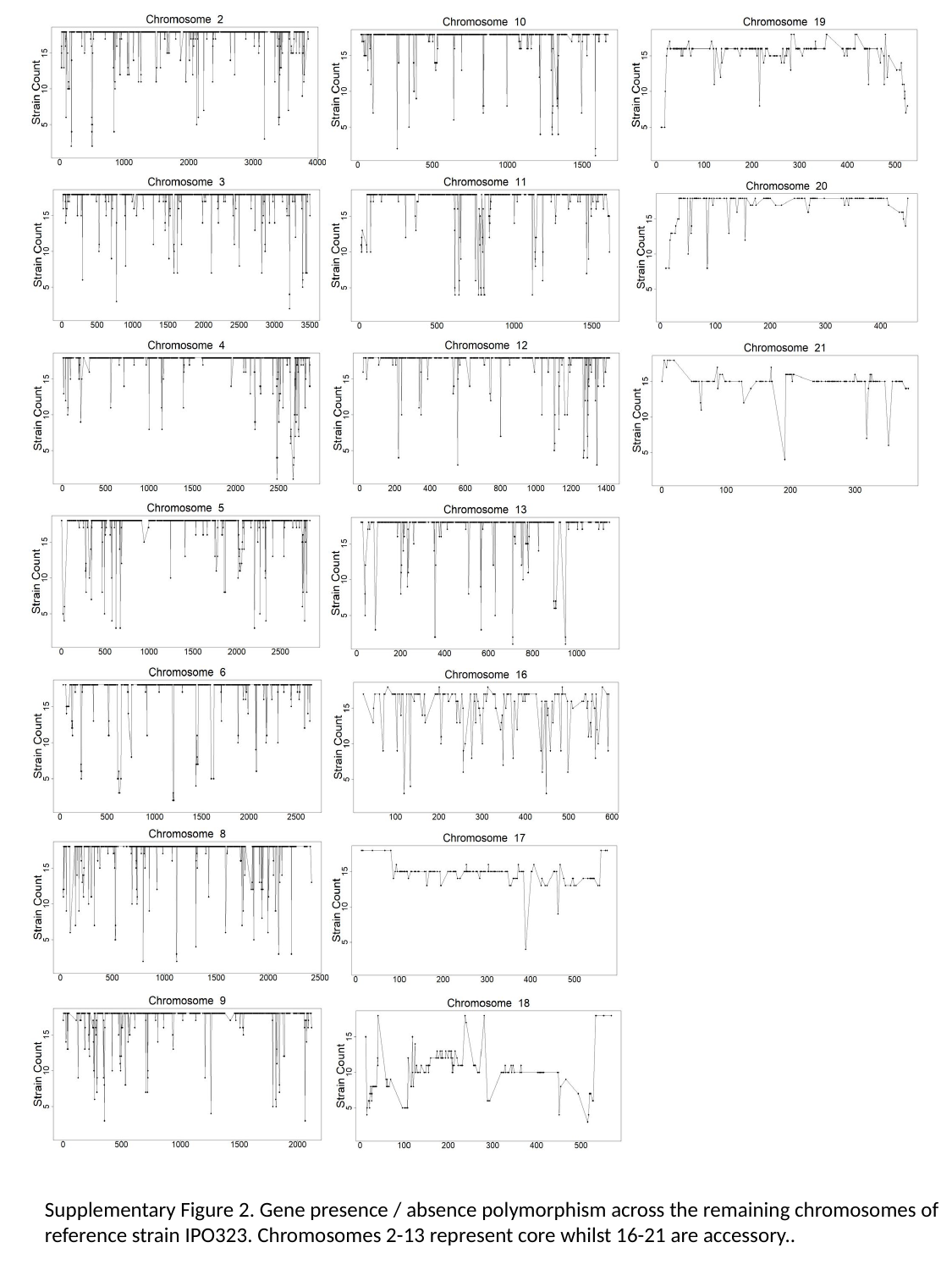

Supplementary Figure 2. Gene presence / absence polymorphism across the remaining chromosomes of
reference strain IPO323. Chromosomes 2-13 represent core whilst 16-21 are accessory..

## Slide 3
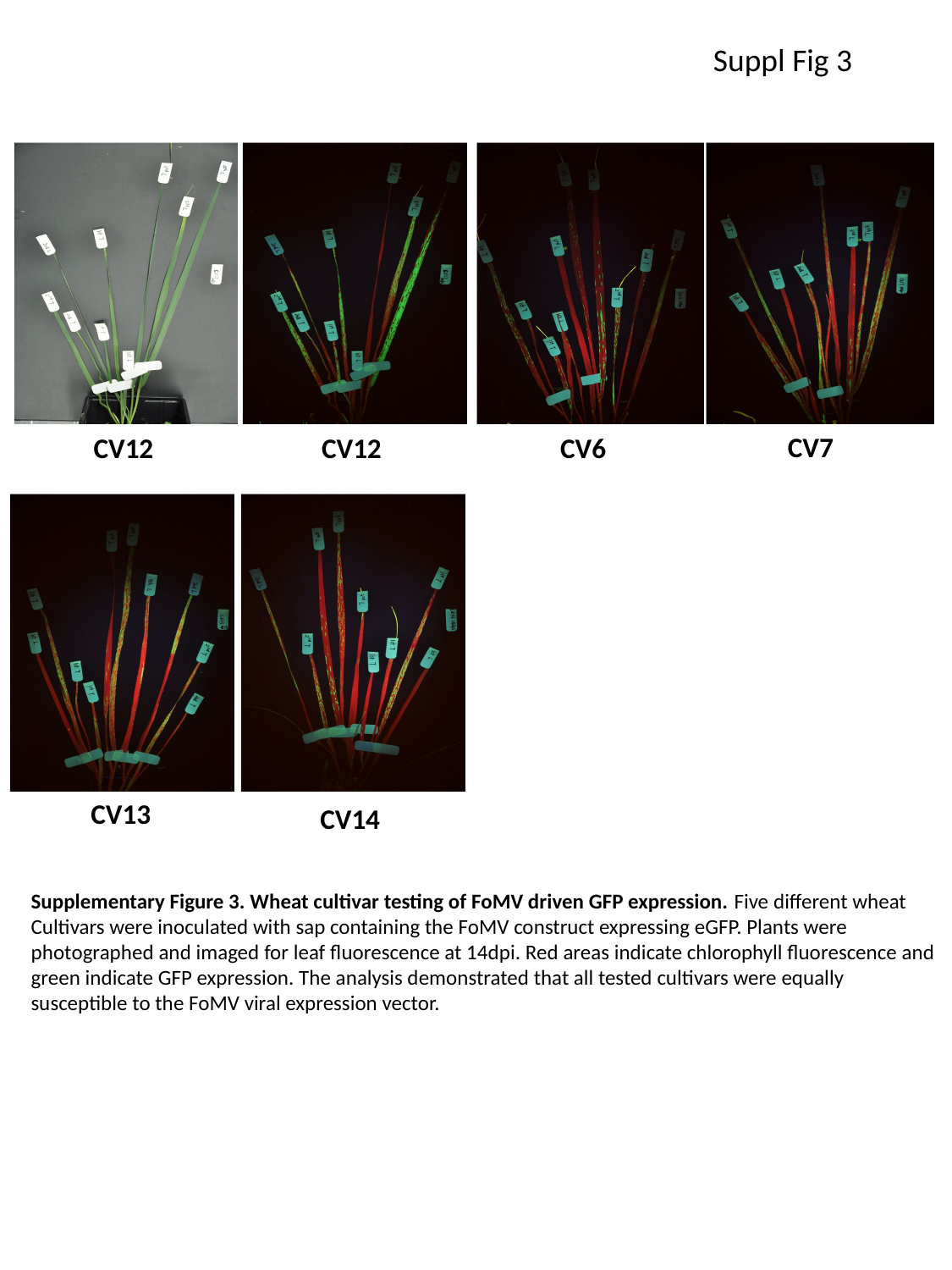

Suppl Fig 3
CV7
CV6
CV12
CV12
CV13
CV14
Supplementary Figure 3. Wheat cultivar testing of FoMV driven GFP expression. Five different wheat
Cultivars were inoculated with sap containing the FoMV construct expressing eGFP. Plants were
photographed and imaged for leaf fluorescence at 14dpi. Red areas indicate chlorophyll fluorescence and
green indicate GFP expression. The analysis demonstrated that all tested cultivars were equally
susceptible to the FoMV viral expression vector.

## Slide 4
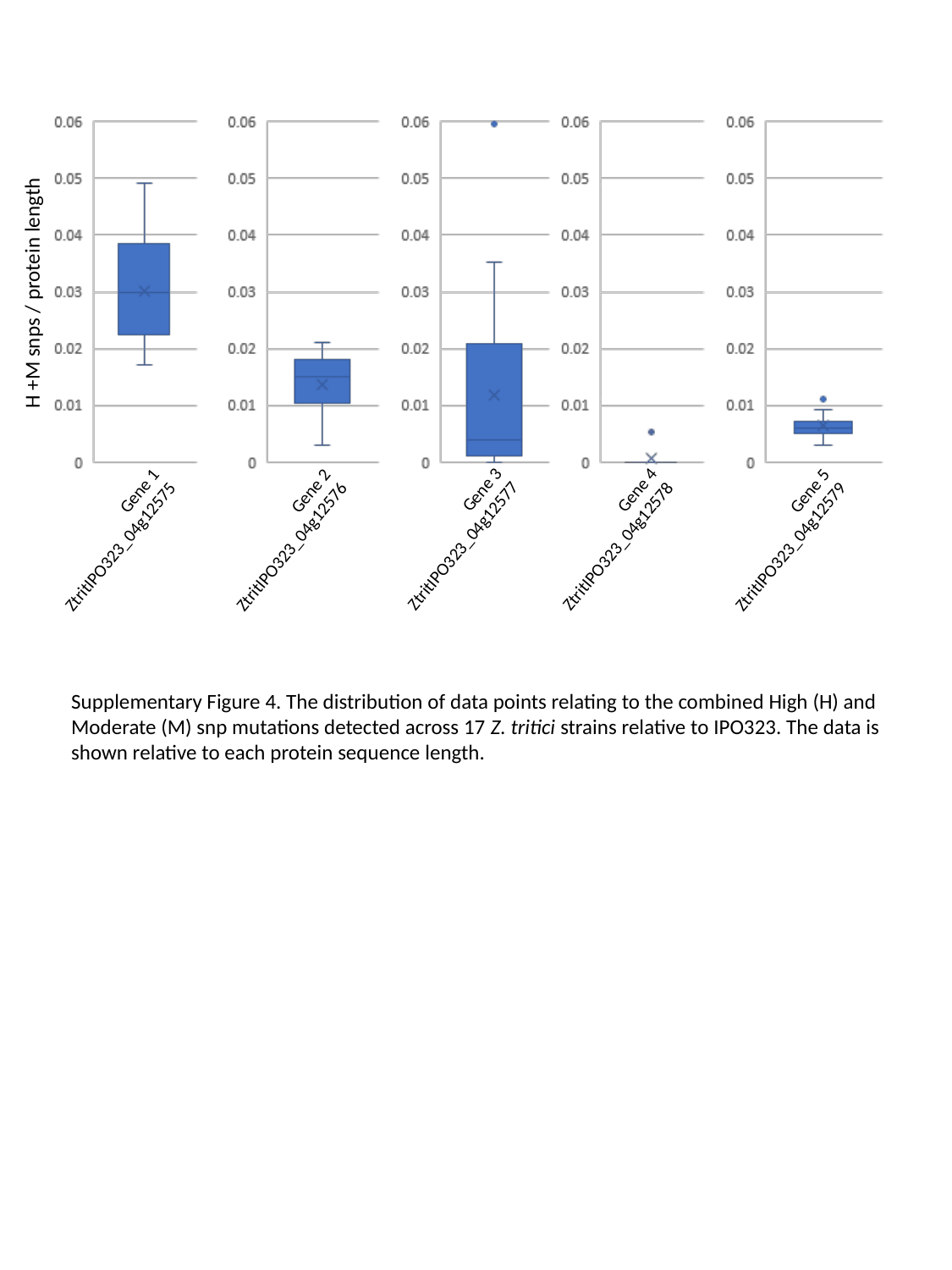

H +M snps / protein length
Gene 3
ZtritIPO323_04g12577
Gene 4
ZtritIPO323_04g12578
Gene 1
ZtritIPO323_04g12575
Gene 5
ZtritIPO323_04g12579
Gene 2
ZtritIPO323_04g12576
Supplementary Figure 4. The distribution of data points relating to the combined High (H) and
Moderate (M) snp mutations detected across 17 Z. tritici strains relative to IPO323. The data is
shown relative to each protein sequence length.

## Slide 5
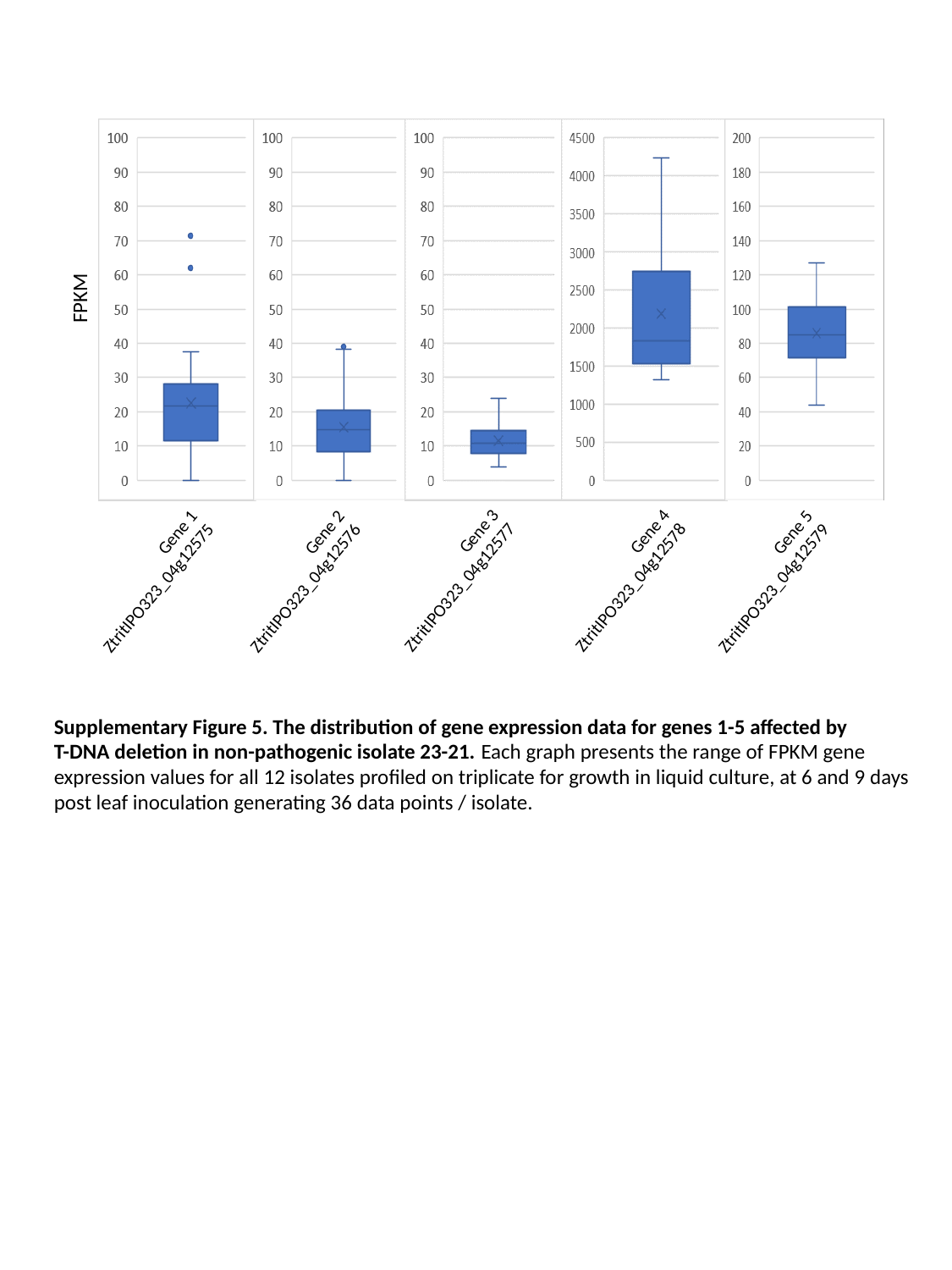

FPKM
Gene 3
ZtritIPO323_04g12577
Gene 4
ZtritIPO323_04g12578
Gene 1
ZtritIPO323_04g12575
Gene 5
ZtritIPO323_04g12579
Gene 2
ZtritIPO323_04g12576
Supplementary Figure 5. The distribution of gene expression data for genes 1-5 affected by
T-DNA deletion in non-pathogenic isolate 23-21. Each graph presents the range of FPKM gene
expression values for all 12 isolates profiled on triplicate for growth in liquid culture, at 6 and 9 days
post leaf inoculation generating 36 data points / isolate.

## Slide 6
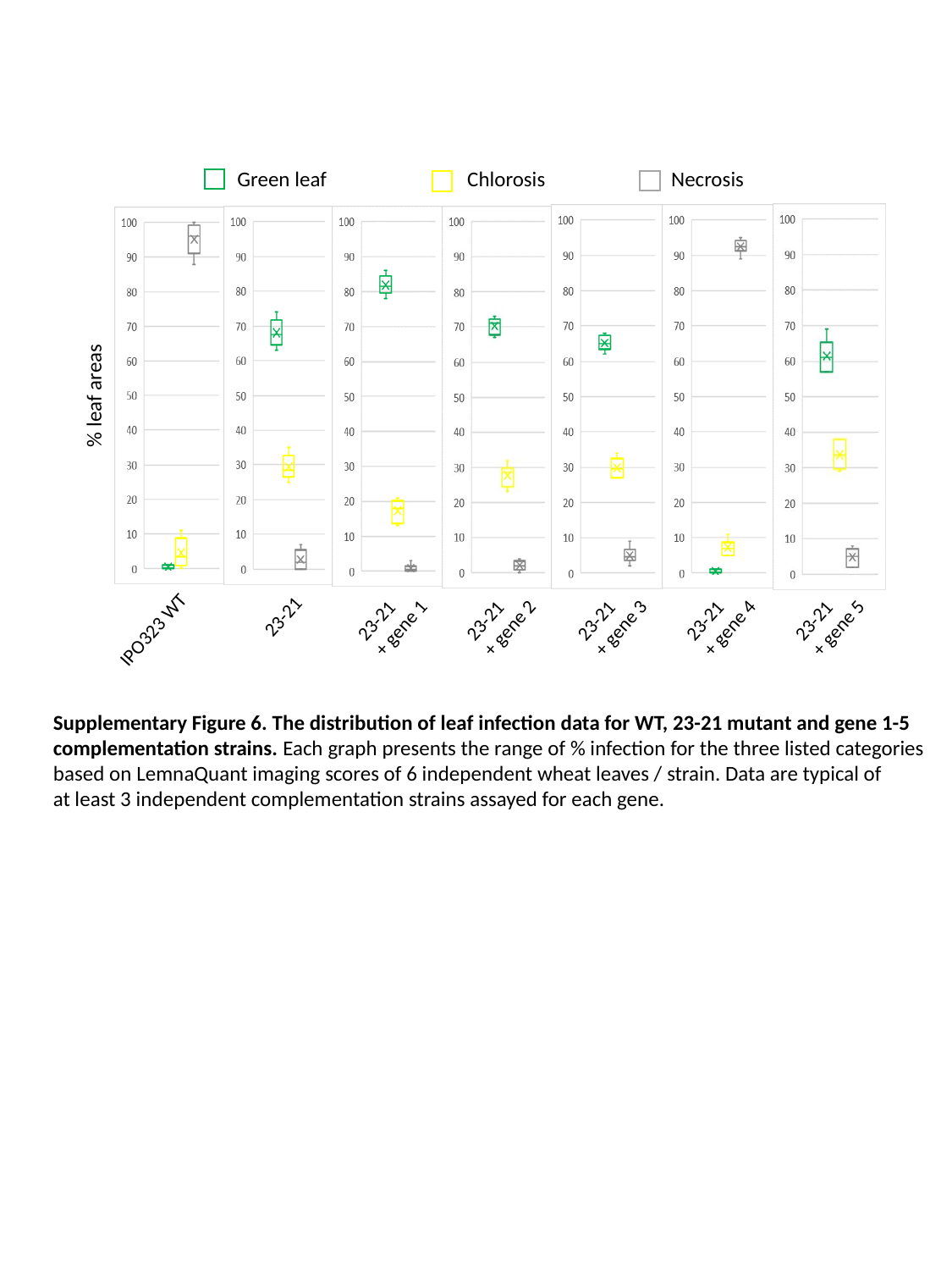

Green leaf Chlorosis Necrosis
% leaf areas
23-21
+ gene 2
23-21
+ gene 1
23-21
+ gene 4
23-21
+ gene 3
23-21
+ gene 5
23-21
IPO323 WT
Supplementary Figure 6. The distribution of leaf infection data for WT, 23-21 mutant and gene 1-5
complementation strains. Each graph presents the range of % infection for the three listed categories
based on LemnaQuant imaging scores of 6 independent wheat leaves / strain. Data are typical of
at least 3 independent complementation strains assayed for each gene.
