# Supplementary material for: Combined pangenomics and transcriptomics reveals core and redundant virulence processes in a rapidly evolving fungal plant pathogen
Source: BMC Biol. 2023 Feb 6;21:24. doi: 10.1186/s12915-023-01520-6 (PMC9903594; doi:10.1186/s12915-023-01520-6)

**IPO323_18S**

**ncrna chromosome:MG2:7:1684914:1686712:1 gene:EFMGRG00000000027 gene_biotype:rRNA transcript_biotype:rRNA gene_symbol:18s_rRNA description:18s_rRNA [Source:RNAMMER;Acc:18s_rRNA]**

1 10 20 30 40 50

| | | | | |

ACCTGGTTGATTCTGCCAGTAGTCATATGCTTGTCTCAAAGATTAAGCCA

TGCATGTCTAAGTATAAGCAACTATACGGTGAAACTGCGAATGGCTCATT

AAATCAGTTATCGTTTATTTGATAGTACCTTACTACATGGATAACCGTGG

TAATTCTAGAGCTAATACATGCTAAAAACCTCGACTTCGGAAGGGGTGTA

TTTATTAGATAAAAAACCAATGCCCTTCGGGGCTCCTTGGTGAATCATAA

TAACTTCACGAATCGCATGGCCTTGCGCCGGCGATGGTTCATTCAAATTT

CTGCCCTATCAACTTTCGATGGTAGGATAGAGGCCTACCATGGTTTCAAC

GGGTAACGGGGAATTAGGGTTCGACTCCGGAGAGGGAGCCTGAGAAACGG

CTACCACATCCAAGGAAGGCAGCAGGCGCGCAAATTACCCAATCCCGACA

CGGGGAGGTAGTGACAATAAATACTGATACAGGGCTCTTTTGGGTCTTGT

AATTGGAATGAGTACAATTTAAATCCCTTAACGAGGAACAATTGGAGGGC

AAGTCTGGTGCCAGCAGCCGCGGTAATTCCAGCTCCAATAGCGTATATTA

AAGTTGTTGCAGTTAAAAAGCTCGTAGTTGAACCTTGGGCCTGGCTGGCC

GGTCCGCCTCACCGCGTGTACTGGTCCGGCCGGGCCTTTCCTTCTGGGGA

GCCGCATGCCCTTCACTGGGCGTGTCGGGGAACCAGGACTTTTACTTTGA

AAAAATTAGAGTGTTCAAAGCAGGCCTTTGCTCGAATACATTAGCATGGA

ATAATAGAATAGGACGTGTGGTTCTATTTTGTTGGTTTCTAGGACCGCCG

TAATGATTAATAGGGATAGTCGGGGGCATCCGTATTCAATTGTCAGAGGT

GAAATTCTTGGATTTATTGAAGACGAACTACTGCGAAAGCATTTGCCAAG

GATGTTTTCATTAATCAGTGAACGAAAGTTAGGGGATCGAAGACGATCAG

ATACCGTCGTAGTCTTAACCATAAACTATGCCGACTAGGGATCGGTGGAT

GTTATCTTTTTGACTCCATCGGCACCTTACGAGAAATCAAAGTTTTTGGG

TTCTGGGGGGAGTATGGTCGCAAGGCTGAAACTTAAAGAAATTGACGGAA

GGGCACCACCAGGCGTGGAGCCTGCGGCTTAATTTGACTCAACACGGGGA

AACTCACCAGGTCCAGACACAAGTAGGATTGACAGATTGAGAGCTCTTTC

TTGATTTTGTGGGTGGTGGTGCATGGCCGTTCTTAGTTGGTGGAGTGATT

TGTCTGCTTAATTGCGATAACGAACGAGACCTTAACCTGCTAAATAGCCA

GGCCCGCTTTGGCGGGTCGCCGGCTTCTTAGAGGGACTATCGGCTCAAGC

CGATGGAAGTTTGAGGCAATAACAGGTCTGTGATGCCCTTAGATGTTCTG

GGCCGCACGCGCGCTACACTGACGGAGCCAACGAGTTCATCACCTTGGCC

GAAAGGTCTGGGTAATCTTGTTAAACTCCGTCGTGCTGGGGATAGAGCAT

TGCAATTATTGCTCTTCAACGAGGAATGCCTAGTAAGCGCATGTCATCAG

CATGCGTTGATTACGTCCCTGCCCTTTGTACACACCGCCCGTCGCTACTA

CCGATTGAATGGCTCAGTGAGGCCTTCGGACTGGCTCAGGGAGGTCGGCA

ACGACCACCCAGAGCCGGAAAGTTGGTCAAACTCGGTCATTTAGAGGAAG

TAAAAGTCGTAACAAGGTCTCCGTAGGTGAACCTGCGGAGGGATCATTA

**IPO323_EF1α_1-1309**

**dna:chromosome chromosome:MG2:4:1:2880011:1 REF**

1 10 20 30 40 50

| | | | | |

ATGGGTGGATACAGTAAGGAGAAGACTCATATCAACGTCGTCGTTATCGG

CCACGTCGACTCCGGCAAGTCGACCACCACCGGACACTTGATCTACAAGT

GCGGTGGAATCGACAAGCGTACCATCGAGAAGTTCGAGAAGGAAGCCGCC

GAGCTTGGCAAGGGCTCCTTCAAGTATGCCTGGGTGCTCGACAAGCTCAA

GGCCGAGCGTGAGCGTGGTATCACCATTGATATCGCACTCTGGAAGTTCG

AGACCCCCAAGTACTACGTAACAGTTATCGACGCCCCTGGTCACCGTGAT

TTCATCAAGAACATGATCACTGGTACTTCCCAGGCCGATTGCGCCATTCT

CATCATTGCCGCCGGTACTGGTGAGTTCGAGGCTGGTATCTCCAAGGATG

GCCAGACTCGTGAGCACGCTCTCCTCGCCTACACCCTCGGTGTGAAGCAG

CTCATCGTCGCCATCAACAAGATGGACACCACCAAGTGGTCCGAGGCTCG

CTTTGGTGAGATCATCAAGGAGACTGCCAACTTCATCAAGAAGGTCGGCT

ACAACCCCAAGACCGTCCCCTTCGTGCCAATCTCCGGATTCAACGGCGAC

AACATGATCGACGTTTCCTCCAACTGCCCCTGGTACAAGGGATGGGAGAA

GGAGACCAAGACCAAGACCACCGGCAAGACCCTACTCGAGGCCATCGATG

CCATCGACCAGCCCGCTCGTCCTTCCGACAAGCCTCTCCGCCTTCCCCTC

CAGGATGTGTACAAGATTGGTGGTATCGGAACAGTTCCCGTCGGCCGTGT

CGAGACCGGTATCATCAAGGCCGGCATGGTCGTCACCTTCGCCCCCGCTG

GTGTCACCACTGAAGTCAAGTCCGTCGAGATGCACCACGAGCAGCTCGTC

GAGGGTGCTCCAGGTGACAACGTCGGATTCAACGTCAAGAACGTCTCCGT

CAAGGAGATTCGTCGTGGCAACGTCGCCGGTGACTCCAAGAACGACCCGC

CAAAGGGTTGTGACTCCTTCAACGCCCAGGTCATCGTCCTCAACCACCCC

GGTCAGGTCGGTGCTGGATACGCCCCAGTTCTGGACTGCCACACCGCCCA

CATCGCCTGCAAGTTCTCTGAGCTCCTCGAGAAGATCGATCGCCGTACCG

GCAAGTCCATCGAGGACCAGCCCAAGTTCATCAAGTCTGGTGACGCCGCC

ATCGTCAAGATGATTCCGTCCAAGCCTATGTGTGTTGAGGCCTTCACTGA

GTACCCACCTCTCGGCCGCTTCGCCGTGCGCGACATGCGTCAAACCGTCG

CCGTCGGTG

**IPO323_ITS1**

**Zt internal transcribed spacer 1**

1 10 20 30 40 50

| | | | | |

CCGAGCGAGGGCCTCCGGGTCCGACCTCCAACCCTTTGTGAACACATCCC

GTTGCTTCGGGGGCGACCCTGCCGGGCGCCCCCGGAGGACCACCAAAAAA

CACTGCATCTCTGCGTCGGAGTTTACGAGTAAATCGAAACA

**IPO323_ITS2**

**Zt internal transcribed spacer 2**

1 10 20 30 40 50

| | | | | |

CACCACTCCAGCCTCGCTGGGTATTGGGCGTCTTTTCGCGGGGGATCACT

CCCCCGCGCGCCTCAAAGTCTCCGGCTGAGCGGTCTCGTCTCCCAGCGTT

GTGGCATCACGTCTCGCCGCGGAGTTCACGAGCCCTCACGGCCGTTAAAT

CACACCTCAGG

**IPO323_RPB1**

RNA polymerase II largest subunit

1 10 20 30 40 50

| | | | | |

ATGGCTACCTTCGCCCACTCGCAGGCGCCGCTGCGTACCGTGCAGGAAAT

CCAGTTTGGTCTTTTCTCGCCCGAAGAAATCAAGAATATGAGTGTCTGTC

ACATCGAGTATCCTGAAACAATGGACGAGCAGCGCAATCGGCCGCGCGAG

AAGGGTCTGAATGATCCGAAGCTCGGCACCATTGACCGGAACACCATGTG

CGCTACTTGCGGTGAAAGCCAGCAAGAGTGTCCTGGTCATTTCGGCCACA

TCGAGCTGGCGGCTCCGGTCTTCCACGTCGGTTTCATTACCAAGATCAAG

AAGATCCTCGAATCGGTGTGCAACAACTGCGGAAAGCTCCTGGAAGATGA

ACGCAATCCTCAGTTTGCGCAGGCCGTGAAGATTCGGGACCCGAAGCGAA

GATTCGAGCAAATCGCAAAGCTGTGCAAGTCAAAGATGGATTGCGCCATG

GACGAGCCCGCTGATGCGAACGACGGCTTCGGTGAAGACCCCAAGAAGCC

CAAGATTGCTGGTCACGGAGGCTGCGGCAATGTTCAGCCGACCATCCGAA

AGGTTCAGCTGACGTTGACCGCGAGTACGAAGGTTGCAAAGTCCGAGGAC

GGCAACGAGGAGGGCGGCGTGGAGAAAAAGACCATCACCCCTCAGGTTGC

CCTCGACCTCTTCAAAAAACTTCGCGAGGAGGATCTGCATCGTCTGGGTC

TGAATGTCGACTACGCTCGACCGGAGTGGATGATCATGACAGTCCTGCCC

GTTCCTCCCCCGGCTGTGCGCCCGAGTATCTCGGTTGATGGCACAAGCCA

GGGTATGCGTTCAGAAGACGACTTGACATACAAGTTGTCTGACATCATTC

GCGCAAACTCCAACGTCCGACGTTGTGAGCAGGAGGGCTTGCCAGCACAC

GTTCGCGAGGAGTTTTTCGGTCTACTCCAATACCACGTTGCCACCTATAT

GGACAACGACATCGCTGGGCTGCCTCGGTCGATTCAAAAGAGTGGGCGCC

CTCTCAAGGCGATTCGCGCACGCCTGAAGTCCAAGGAGGGGCGTCTTCGA

GGTAACTTGATGGGCAAGCGTGTGGACTTTTCTGCTCGAACCGTGATCAC

CGGTGACCCCAACCTAGATCTCGATCAAGTTGGAGTGCCTAGGTCGACCG

CTCGAGTCTTGACCTTTCCGGAGCGCGTCACAGTCTACAACATCCACAAG

ATGCAAGAACTTGTTCGCAATGGACCGGACCAGCACCCCGGGGCTAAGCA

CGTCATCCGCGAGGATGGCTCTCGTATCGACTTGCGATACCACAAGCGTG

CGGGTGAGATTCAGCTCCAGCTCGGCTGGATTGTCGAGCGACACATCGTT

GATGGAGATTACATCATCTTCAATCGACAGCCGTCGCTGCACAAGGAGTC

CATGATGGGTCATCGAGTCAAGGTCATGCCTTACTCCACCTTCCGCATGA

ATCTCTCAGTCACATCGCCATACAACGCCGACTTTGACGGTGACGAGATG

AACTTGCACGTGCCCCAAGGACACGAGACCCGCGCGGAGGTCGCCAATCT

CTGCGCCGTGCCACACAACATCGTCTCTCCGCAGAAGAACGGACCTCTGA

TGGGTATTGTGCAAGATACCATGGCTGGCTGCTGGATGATGACGAAGAAA

GATGTCATGATCGACTACCAAGAGCTGATGAACATTCTGCTATGGGTGCC

TTCGTGGGATGGTGTAGTTCCGCCTCCCGCCATCATCAAGCCACAGCCTC

GATGGACTGGCAAGCAAGTGGCATCGCTCTTCTTCCCCCCTGGTCTCAAC

TACTTCATGCCCGCATCCAAGAACGACGACAACCCGCACGAGGAGAAGAA

GGAGATTCTCGTCCAGAATGGCGAGATCATGTGGGGCCGAATCTGGAAGC

AGGTGGTTGGTGCTTCGCAGAGTGGTGTTGTTCACTACATCTTCAACGAT

CGCGGTCCCGAGGCAGCGGTCGAGTTCTTCAGCGGGTGCCAGCGCATTGT

CTGCCACTGGATGCTTCATCACGGCTTCAGTGTAGGCATCGGTGACACCA

TCCCGGATGATCACATGGTGGGCGAGATCGAAGGCGCCATTGTCGAAGAG

AAGCTTCAAGTCGACAAATACGTGGAACAGGTGCAGTCAGACGCCATGGA

GACACTGCCTGGTATGACAATCCGCGAGACTTTCGAGTCGCAGACCAAGG

CTGCCCTCGACAATGCTCGAAACAAGGCTGGTGACGCAGCTTTCGCCCTG

ATGAAGTCGTGCAACAATGTCGGAGTCATGGTCAACTCGGGCTCCAAGGG

TTCCAGCACGAACGTCTCCCAGATGACCGCCGCAGTGGGACAGCAGTCTC

TCGAGGGCAAGCGATTGCCGTTCGGCTTCAAATACCGTGTGTTGCCCCAT

TTTCCAAAGGACGATTACTCGCCGGCATCACGAGGATTTGTCGAGAATTC

TTATCTTCGTGGCCTCACCCCCCAGGAGTTCTTCTTCCACGCCATGGGTG

GTCGAGAAGGTCTGATTGATACCGCTGTCAAGACTGCCGAGACTGGATAC

ATTCAGCGTCGTCTTGTCAAGGCGCTGGAGGAGATCATGATCAAGTACGA

TGGCACCGTTCGAAACTCGCTCGGCGACATCCTGCAATTCACGTACGGAG

AAGACGGTCTCGATGCGACATACATCGAGTCCCAGCACATCAACACGATC

GCCTCCTCGCACGGGCAGTTTGATCGCAAGTACAAAATCGACGTCATCAG

CCAGAACAAGGAATTCGCGCTGACTCCTGCCAACCTGGAAATGGCTGCCG

AGCTCATTGGCGATGTCGAAATCCAGCAGCTGTTCGACCAAGAGTACGAG

GCCATCAGCAATGACAGGCAGAAGATTCGAAATGGTCTTGACGATCCTGA

AGAGAAGCGCTACTTGCCACTGAACATCAATCGTATGATTCAGAACGCGC

GGAATAAGTTCAAGATCAACGATAACAGCAAGAGCGACCTTGACCCACGG

GAGACCATTCCCAAGATTCAAGCCTTGCTCGACCGCCTCATCGTCATTCG

CGGTGACGACTCCTTGTCTCAGGAGGCAGACATGAACGCCACACTTTTGT

GCAAGGCCATGTTCCGCTCTCGCTTGGCATTCAAGCGTCTGGTCAAGGAA

GATAAGCTCAACAAGCTTGCGCTTGACAATGTCCTTGGCGATCTTGAGAA

CAGATTCCTGCGTGCTCTTGTCAACCCTGGTGAGATGGTGGGTGTTCTCG

CAGCTCAGTCGATTGGCGAACCCGCTACGCAAATGACGCTCAACACTTTC

CATTTGGCTGGTGTGACTGCAAAGACTACAACCAAGGGTGTGCCTCGTCT

CAAAGAAATTCTCAACGTTGCCGAGAACATCAAGACGCCCAACATGAAAG

TGTTCCAGGATCCAGCGCACGGTCTCACGCAGGAAGGAGCCAAGAATCTT

CGGTCCATGATCGAGCACACCAGTCTGCGCAACGTCACGGACGTCACTGA

GATCTACTATGATCCAGTCATCGAAGACACTGTCATTCAAGCTGACCACG

ACATGGTTGAGTCGTACTTTATCATTCCGGAAGAGAGCGAGCGGCCTGAA

TTGCAGAGCAAGTGGTTGCTCCGTATTGTGCTTGGTCGTCGCCAGCTTCT

CGATAAGGGCTTGACTGTGACCAATGTGGCCGACAAGATCAAGGAGGTTT

TCGGCGGAGATGTTGCGGTCATCTTCTCCGATGACAACGCCGATGAGCAA

GTCATCCGTGTTCGCATGATCACTGGCGGACGCGACAAGGAGAATGAGTC

CGAAGAGGAGCAAGAAGACACCCTCAAGCGCCTTGAGGGACACATGCTGG

ACACTGTCGTGCTTCGTGGAGTGTCCGGCGTCAAGCGTGCTTTCGTCTCG

AACGAAGCGAGAATGATCACAATCGAAGATGGCTCGCTTGTCAAGAGCAA

CAGCGTCGAGGCATGCAAGGAGTGGCTCCTCGACACTGATGGTGTGAACC

TCAAGGACGTCTTGGCAGTTGAGGGCGTTGATTCCACCCGCACGACTTGC

AATCACTTCCAGACCATCATGAAGGTGTTTGGTATTGAGGGTGTTCGGGC

ATCGTTGATCAAGGAGTTCAAGGATGTGCTGACCAACGACGGTTCCTACG

TCAACCACCGCCACATGGCCATCCTCTGTGATGTGATGTGTGCGCGAGGA

GAGCTGATGGCTGTCACTCGACATGGTATCAACCGTGCCGACACTGGCGC

GCTGATGCGTTGTTCTTTCGAAGAGACTGTCGAGATCTTGTTTGACGCTG

CCTCATCTGGCGAGCTCGACGACTGCCGCGGTGTGTCCGAGAACATCATT

CTGGGACAACTTGCGCCCAGCGGTACTGGAGAATTCGACATGCTCCTCGA

CACGGAGATGCTCAAGTCAGTCGCACCAACTCAGCGTGCTATGCATTCTG

GTATCGGTGTCGGTGCTGGCTCTCCCGAGGGCGCCATGACGCCTTACGAC

ATCGGCTCGCCTTTGGCAGACGGCGGTTACGCAGGTCCTGACTACGGAGC

CAGCTTCTCACCTATCATCAACCCTGGTCAGGACGAGGGTGGTGGCCTCA

CTGCTTACGGCGGCGGCTTCGATGGTGGTATGAGCCCGTACCGCGGCGGC

ATGAGCCCTGGCTATGCTCCCACGAGTCCGTTCAACAGTGGAGGCTTTTC

GCCCACTTCGCCTGCCTATGGCTACTCTCCCACCTCTCCTGGCATGGCTG

GATACTCGCCGACCTCTCCTGGCCAAGGCATGACCAGCCCGGCGTATCAA

GTCACATCTCCCCAGTTCTCACCCGCGAGCCCAGCCTATACGCCAACATC

ACCGACGTATTCGCCCACATCGCCAGCATACAGCGGTGGCAGCAAGATTT

CTCCCACATCGCCATCCTACTCACCGACATCGCCGTCGTACTCCCCAACC

TCTCCCTCATACTCTCCTACATCACCCAACTACAGCCCGACCTCACCTGC

GCATGCCCCTGGCAGCGCTACCTCTCCGAAGTACTCCCCAACTTCACCGA

CGTACAGTCCGACGTCACCCGCATACAGCCCGACGTCGCCGACTTACTCG

CCGACAAGTCCGAAGTATGGTAGCGGTGTTGGCGCGAGCGGTGCCTCGCC

GACTTCGCCGACATACAGCCCAACATCGCCGGTGTACAGCCCGACCAGCC

CTGCTCAGAATGGGTACTCACCAACCTCGCCCGGGCAGAAGCAGAGCCCG

ACGAGCCCGCAGTACAGCCCAACCAGCCCGCAATACTCTCCGAACTCGCC

GCAAGATAACTCAGGATCGTGA

**IPO323_RPB2**

RNA polymerase II second largest subunit

1 10 20 30 40 50

| | | | | |

ATGGGCGACTTCGGTGGAGATGCGATGATGGCGGAGGAGGAAGGCACGTG

GGACGACAGCACCATGATCACGGCCGAGGATTGCTGGACGGTTATCCACT

CTTTCTTCGATGAAAAGGGTCTGGTCTCCCAGCAATTGGACTCGTTCGAT

GAATTCGCTAGCACGACAATGCAGCAGATCGTGGACGACCAGCCAGCGAT

TGTGATTGATCAGAACCTGGCTGGACTGGACGAAGAAAGCGGTATGCCGA

TCGTGAAGAAGCGCTCGAGTATCAAGCTCGGGACTCTTACGGTCAGCCAA

GCAGCCATGACCGAGGGCGACGGTAGCACGAGGGCCATGCACCCTCACGA

GGCTCGTCTACGGAATTTGACTTATTCATCACCCATGTTCATCAAGCTGG

AGAAGACAACACAACTGGCGCGAGAAAGAGCACTGGGTGGACACTACGAC

GAGGATCAGGGCATGTGGGTAGCGCCACCGAACTGGGACGGCACAGTGGA

GACGGTCTGGGAGGAAGACCCCGACAATCCCAAGCAGGCTATCGATCAAG

TCTTCATTGGAAAGCTGCCAGTCATGCTGAAGTCGAAGATCTGCGCATTG

AGGAATCGAAGCGAAGCAGAGCTTTATGCTTTCCAAGAATGCCCATTCGA

TCAGGGAGGATATTTCATCATCAACGGTAGTGAGAAGGTGTTGATCGCAC

AAGAGCGGAGTGCTGCAAACATCGTTCAGGTTTTCCGAAAGAAGGGCACA

AACACACCGGTTGTGGCGGAGCTGCGAAGTGCCGTCGAGCGAGGAACACG

CTTGATCAGCTCGATGCAGGTCAAGCTCTGCAATCAAGCAGTGGCAGCTC

AGCATTCTGGACAGACCATTAAAGTCTCCCTCCCATACATCAGGGCCGAT

GTGCCCATCGCTATTGTTTTCCGTGCGATGGGTGTAGTTTCCGACGAGGA

TATCCTGGCACATATCTGTCCAGAGGAGGATACACAAATGCGAGAGAAGC

TCAAGCCTTGTTTGGAAGAGGCATTCGTGATTCAGGACCATGACGTCGCG

CTGGATCATATTGGAAGACGTGGACAGCAGCAGGGCACCAAGGATCGCCG

TATCCGATACGCACGAGATATCATGCAAAAGGAGTTCTTGCCTCACATCT

CGCAAGAAGAGGGCAGTGAGACGAAGAAGGCGTTCTTCCTGGGATACATG

GTCAATCGCATGCTTCAGTGTGCGCTTGGACGGACTGAGGAAGACGATCG

TGATCATTTCGGAAAGAAGCGTCTCGATTTGGCAGGACCTTTGATGGCGC

AGGTTTTCCGTTTGAAGTTCCAACAGTTGGTGAAGGACATGAGGCAGTAC

CTCCACCGATGCGTGGAGACTGGTAAGGACTTCAACATTGCGCTCGCGGT

CAAGACAAATATCATCACATCGGGTTTGCGATACTGCCTGGCGACTGGAA

ATTGGGGTGACCAAAAGAAGGCCGCAAGCGCAAAAGCTGGTGTGAGTCAA

GTGCTGAACAGATACACCTACGCCTCGACCTTGAGTCATTTGCGGCGAAC

GAACACCCCCATCGGTCGTGACGGCAAGATTGCGAAGCCGAGACAACTGC

ACAACACTCACTGGGGTCTCGTCTGCCCGGCCGAAACGCCCGAAGGACAA

GCTTGTGGTCTGGTAAAGAACTTGTCTCTGATGTGCTATGTAACCGTTGG

AACGCCCGGCGAGCCTATCGTTGACTTCATGCGGCAACGTGGAATGGACC

TTCTCGAGGAACACGATCCGAACCAGGTCAAGGATGCGACCAAGGTCTTC

CTGAACGGTACCTGGGTGGGTGTACACAAAAGTGCCGGACAGCTCACAGA

GACTTTACGGCAACTCCGACGAAAGGGTCTTCTCAGTTTCGAAGTCACCA

TCATTCGAGACGTGCGCGAGCGCGAAATTCGAGTCTTCACAGACGCTGGA

CGTGTCTGCCGGCCTCTTTTCGTGGTGGACAACAACCCCAAATCATTGGA

GCCGGGCACTCTTATGCTGAAACAAGATCATGTTGAGAGACTTCACCAAG

ATCAAGAACTTCTCGCCTCGCTTGAAGGCGTTAGTGAAGAAGATCGCGAG

CAGCAGCTCTTCGGTTGGAAGGGTCTGGTCACCAGCGGAGTGGTCGAGTA

CCTTGACGCTGAGGAAGAAGAAGTCGCCATGATCATCATGACGCCTGAGG

ATCTTGAAGAGCACAGAGTCATGCGCAATGGCATGGCCGTCGAGGAACTC

AAGGTCGACCCGCATCGACGGATCAAGCCGAAACCGAACCCACAAGTCCG

GACATACACACATTGCGAGATTCATCCTGCGATGATTCTGGGCATTTGTG

CCAGCATCATCCCCTTCCCAGATCACAACCAGAGTCCTCGCAACACGTAT

CAGGCAACCATGGGTGTCACGTTGACAAACTACAACGTACGAATGGATAC

CATGGCAAATGTCCTCTACTATCCACAAAAACCTCTTGCAACCACACGAA

GTATGGAGTTCTTAAAGTTTCGAGACTTGCCCGCCGGCCAGAATGCCATT

GTGGCGATTGCTTGCTACTCCGGTTACAACCAGGAAGATTCCGTGATCAT

GAACCAAAGCAGTATCGATCGTGGTCTCTTCAGATCGCTGTTCTACCGCG

CGTACATGGACCAGGAGAAGCGCGTGGGCATGAGTGTGGTCGAGTCCTTC

GAGAAGCCCACCCGATCGGATACCATGCGCATGCAACACGGCACATACGA

CAAGCTAGACGAAGACGGCATCATCAGTCCTGGTGCTCGTGTATCCGGTA

CTGACATCCTTATCGGAAAGACCGCGCCTATGCCTCCAGATGCCGAGGAG

CTCGGACAGAGAACGAAACTCCACGTCAAGCGCGATGTCAGCACACCCTT

GAGATCGACTGAGAACGGTGTGGTCGACCAAGTACTTCTCACAACCAACA

CCGAAGGTCTGCGATTCGTCAAGGTCCGAACACGAGTCACGAAAGTGCCA

CAAATCGGAGACAAGTTCGCCTCTCGTCACGGACAAAAGGGTACCATCGG

TATCACCTACCGCCAAGAAGACATGCCTTTCTCCGCAGATGGTCTCACAC

CCGACATCATCATCAATCCCCACGCCATTCCCTCCCGTATGACAATCGCC

CATTTGATCGAGTGTCTCCTCTCCAAGGTCGGCGCCCTCCAAGGCCAAGA

AGGTGACGCCACACCTTTCACCGAAGTCACTGTCACCTCCATCTCTGAGA

TCCTGAAGTCCAACGGCTTCCAACAGCGTGGATTCGAAGTCATGTACAAT

GGCCACACCGGCAAGAAATTGAATGCGCAGGTCTTCCTCGGTCCGACATA

CTACCAACGTCTACGCCACATGGTCGACGACAAAATCCACGCTCGTGCCC

GTGGACCGCTACAGATCCTGACCCGACAACCTGTGGAAGGTCGCGCGAGA

GACGGTGGCCTGCGTTTCGGAGAGATGGAGCGCGATTGCATGATCGCCCA

TGGCGCCAGCGCCTTCCTCAAGGAGCGACTCCTGGACGTGTCGGATGCCT

TTCGCGTGCACATCTGTGAGCTGTGTGGTCTCATGACGCCCATTGCGAGT

ATCAAGAAACAGCAGTTTGAATGTCGTCCATGCAAGAACAAGACGAAGAT

TGCGCAGATCATCATTCCGTATGCAGCGAAATTGCTGTTCCAGGAGTTGG

CGGCGATGAATGTGGCGACGAGGATGTTCACGACGAGGAGTGGGGTTAGC

ATTCGGTAG


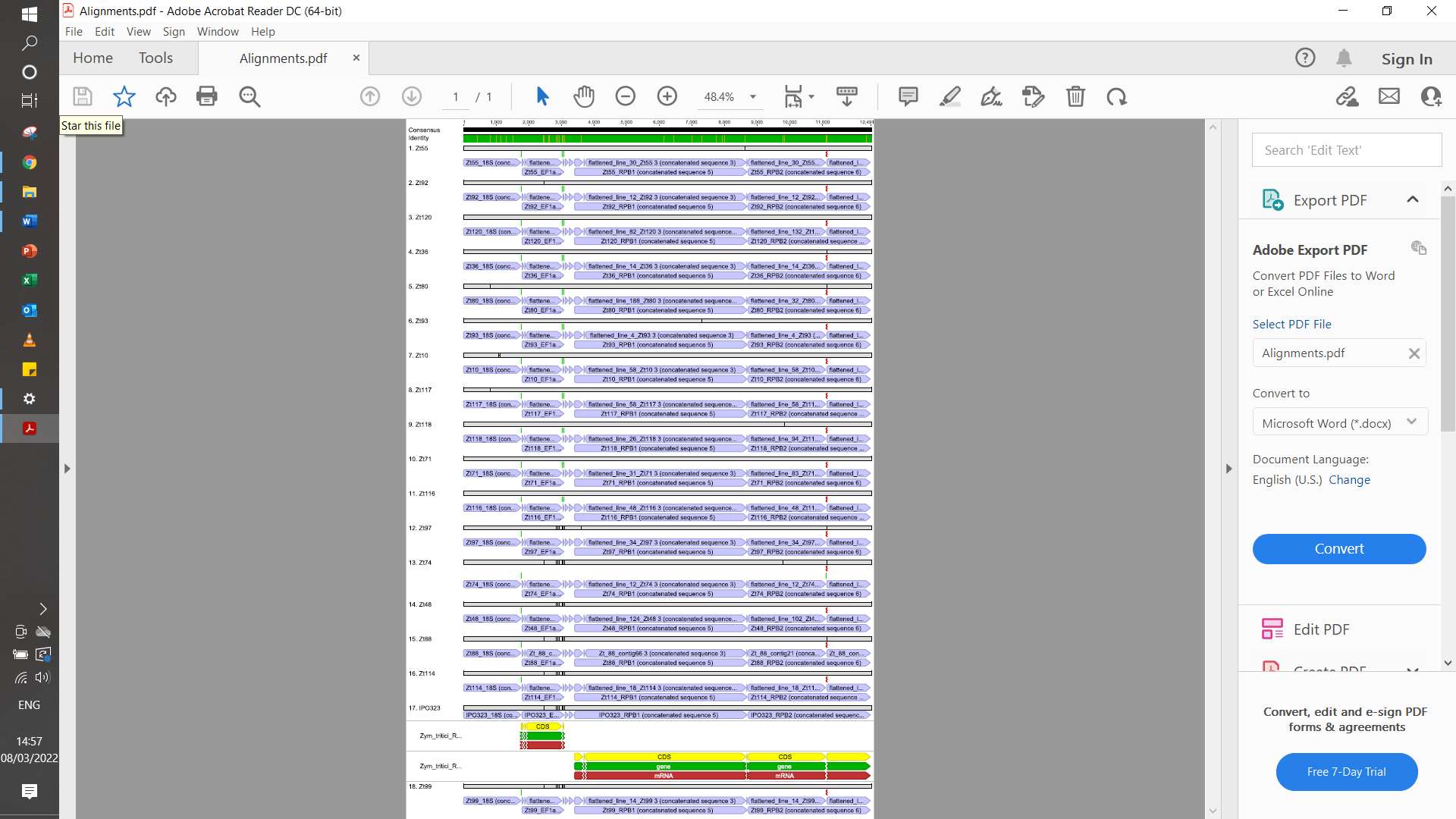

Supplement: Supplementary file 3 — Additional file 3: Data S1. The reference sequences used for phylogenetic analysis of Z. tritici isolates. [file 12915_2023_1520_MOESM3_ESM.docx]
