# Supplementary figures and images for: Combined pangenomics and transcriptomics reveals core and redundant virulence processes in a rapidly evolving fungal plant pathogen
Source: BMC Biol. 2023 Feb 6;21:24. doi: 10.1186/s12915-023-01520-6 (PMC9903594; doi:10.1186/s12915-023-01520-6)

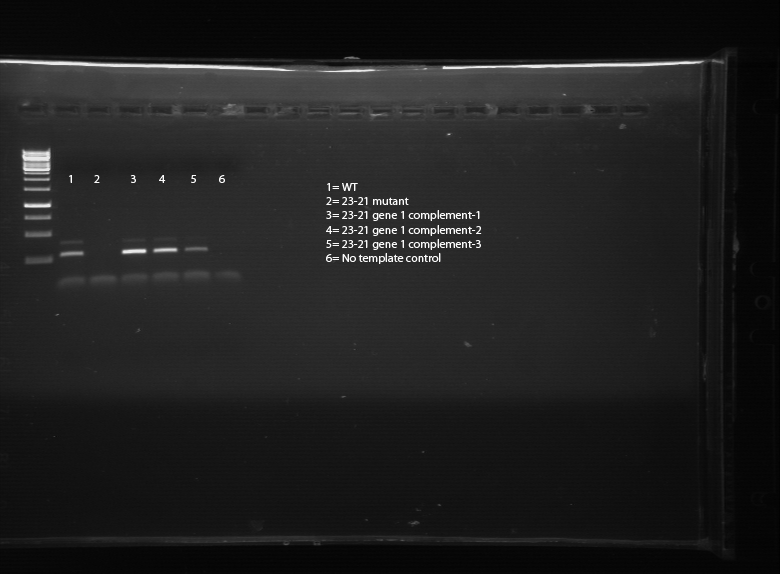

Supplement: Supplementary file 11 — Additional file 11: Data S4. Original gel for RT-PCR analysis on gene 1 complementation strains in 23-21. Note all oligos used span introns. [file 12915_2023_1520_MOESM11_ESM.tif]

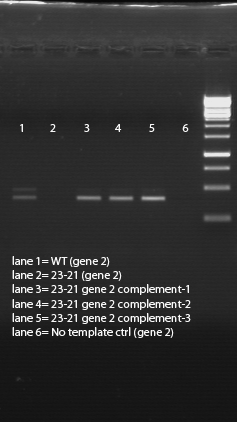

Supplement: Supplementary file 12 — Additional file 12: Data S5. Original gel for RT-PCR analysis on gene 2 complementation strains in 23-21. Note all oligos used span introns. [file 12915_2023_1520_MOESM12_ESM.tif]

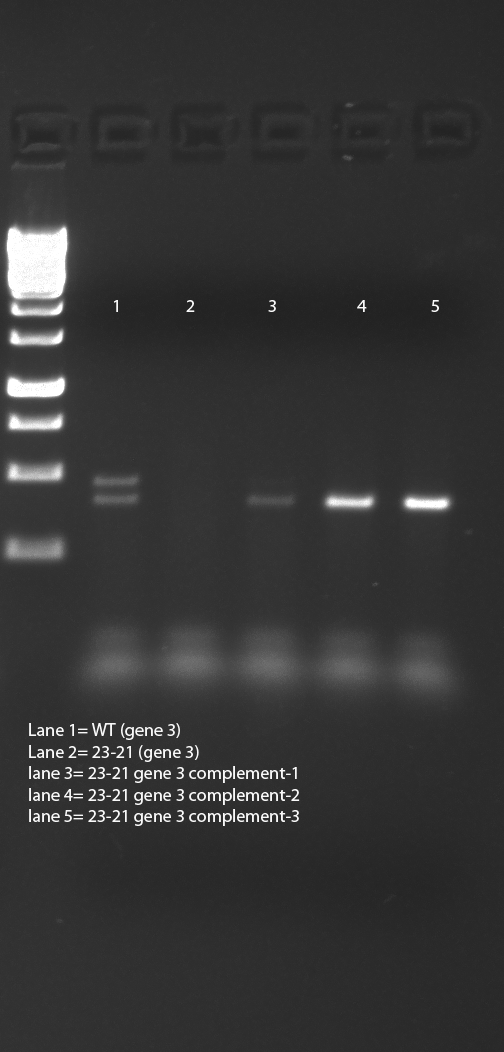

Supplement: Supplementary file 13 — Additional file 13: Data S6. Original gel for RT-PCR analysis on gene 3 complementation strains in 23-21. Note all oligos used span introns. [file 12915_2023_1520_MOESM13_ESM.tif]

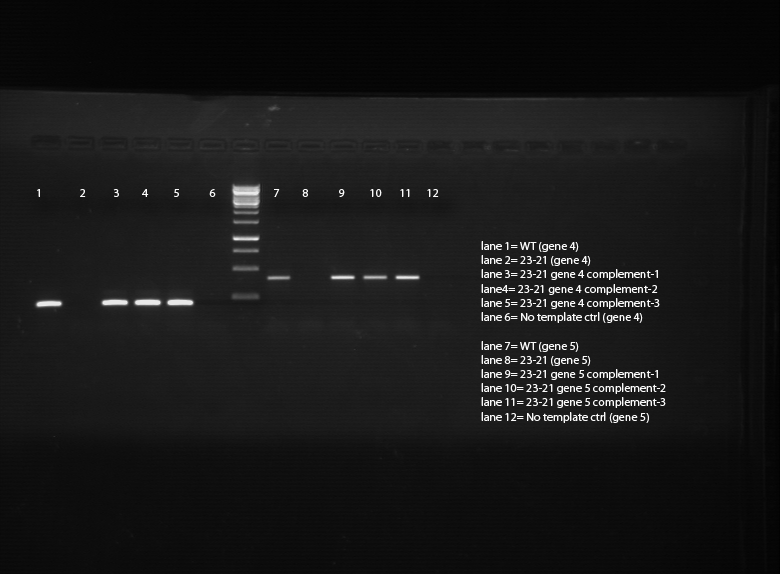

Supplement: Supplementary file 14 — Additional file 14: Data S7. Original gel for RT-PCR analysis on gene 4 and 5 complementation strains in 23-21. Note all oligos used span introns. [file 12915_2023_1520_MOESM14_ESM.tif]
