# Supplementary material for: Combined pangenomics and transcriptomics reveals core and redundant virulence processes in a rapidly evolving fungal plant pathogen
Source: BMC Biol. 2023 Feb 6;21:24. doi: 10.1186/s12915-023-01520-6 (PMC9903594; doi:10.1186/s12915-023-01520-6)
Supplement: Supplementary file 15 — Additional file 15: Data S8. Full Inventory of all data linked to this project. [file 12915_2023_1520_MOESM15_ESM.docx]

**An Inventory of data stored in publicly accessible repositories to support the paper: *Combined pangenomics and transcriptomics reveals core and redundant virulence processes in a rapidly evolving fungal plant pathogen***

**Overview**

The primary raw data is DNA and RNA sequencing. Short-read Illumina DNA sequences were used to assemble 17 *Zymoseptoria tritici* genomes and the gene expression of these genomes in-vitro and in-planta at several time points was assessed by Illumina RNA sequencing.

A pangenome gene sequence and annotation was derived from a comparison of the seventeen new genomes with the reference genome IPO323. The RNAseq data was mapped to the pangenome coding sequences, and a gene expression count file was generated.

All the raw and derived data that the work is based on is archived in public repositories, accessible after publication, using the NCBI BioProject code PRJNA890236 (<https://www.ncbi.nlm.nih.gov/bioproject/PRJNA890236>) and the Rothamsted repository.

The files are listed below:

**Raw Data: DNA Sequences**

| BioProject | Accession | Study | biosample | filename | filename2 |
| --- | --- | --- | --- | --- | --- |
| PRJNA890236 | SRR22188307 | SRP406242 | SAMN31282227 | Zt10_2227_LIB27062_LDI24041_TGACCA_L001_trimP_R1.fq.gz | Zt10_2227_LIB27062_LDI24041_TGACCA_L001_trimP_R2.fq.gz |
| PRJNA890236 | SRR22188306 | SRP406242 | SAMN31282228 | Zt36_FCHMTW3BCXY_L2_wHAXPI055060-94_1.fq.gz | Zt36_FCHMTW3BCXY_L2_wHAXPI055060-94_2.fq.gz |
| PRJNA890236 | SRR22188298 | SRP406242 | SAMN31282229 | Zt48_FCHMTW3BCXY_L2_wHAXPI055061-96_1.fq.gz | Zt48_FCHMTW3BCXY_L2_wHAXPI055061-96_2.fq.gz |
| PRJNA890236 | SRR22188297 | SRP406242 | SAMN31282230 | Zt55_2227_LIB27064_LDI24043_GCCAAT_L001_R1.fq.gz | Zt55_2227_LIB27064_LDI24043_GCCAAT_L001_R2.fq.gz |
| PRJNA890236 | SRR22188296 | SRP406242 | SAMN31282231 | Zt71_2227_LIB27065_LDI24044_CAGATC_L001_R1.fq.gz | Zt71_2227_LIB27065_LDI24044_CAGATC_L001_R2.fq.gz |
| PRJNA890236 | SRR22188295 | SRP406242 | SAMN31282232 | Zt74_FCHMTW3BCXY_L2_wHAXPI055062-97_1.fq.gz | Zt74_FCHMTW3BCXY_L2_wHAXPI055062-97_2.fq.gz |
| PRJNA890236 | SRR22188294 | SRP406242 | SAMN31282233 | Zt80_FCHMTW3BCXY_L2_wHAXPI055063-100_1.fq.gz | Zt80_FCHMTW3BCXY_L2_wHAXPI055063-100_2.fq.gz |
| PRJNA890236 | SRR22188293 | SRP406242 | SAMN31282234 | Zt88_FCHMTW3BCXY_L2_wHAXPI055064-103_1.fq.gz | Zt88_FCHMTW3BCXY_L2_wHAXPI055064-103_2.fq.gz |
| PRJNA890236 | SRR22188292 | SRP406242 | SAMN31282235 | Zt92_FCHMTW3BCXY_L2_wHAXPI055065-107_1.fq.gz | Zt92_FCHMTW3BCXY_L2_wHAXPI055065-107_2.fq.gz |
| PRJNA890236 | SRR22188291 | SRP406242 | SAMN31282236 | Zt93_FCHMTW3BCXY_L2_wHAXPI055066-108_1.fq.gz | Zt93_FCHMTW3BCXY_L2_wHAXPI055066-108_2.fq.gz |
| PRJNA890236 | SRR22188305 | SRP406242 | SAMN31282237 | Zt97_2227_LIB27066_LDI24045_CTTGTA_L001_R1.fq.gz | Zt97_2227_LIB27066_LDI24045_CTTGTA_L001_R2.fq.gz |
| PRJNA890236 | SRR22188304 | SRP406242 | SAMN31282238 | Zt99_FCHMTW3BCXY_L2_wHAXPI055067-32_1.fq.gz | Zt99_FCHMTW3BCXY_L2_wHAXPI055067-32_2.fq.gz |
| PRJNA890236 | SRR22188303 | SRP406242 | SAMN31282239 | Zt114_2227_LIB27069_LDI24048_ACTTGA_L001_R1.fq.gz | Zt114_2227_LIB27069_LDI24048_ACTTGA_L001_R2.fq.gz |
| PRJNA890236 | SRR22188302 | SRP406242 | SAMN31282240 | Zt116_2227_LIB27070_LDI24049_GATCAG_L001_R1.fq.gz | Zt116_2227_LIB27070_LDI24049_GATCAG_L001_R2.fq.gz |
| PRJNA890236 | SRR22188301 | SRP406242 | SAMN31282241 | Zt117_FCHMTW3BCXY_L2_wHAXPI055068-35_1.fq.gz | Zt117_FCHMTW3BCXY_L2_wHAXPI055068-35_2.fq.gz |
| PRJNA890236 | SRR22188300 | SRP406242 | SAMN31282242 | Zt118_2227_LIB27071_LDI24050_TAGCTT_L001_R1.fq.gz | Zt118_2227_LIB27071_LDI24050_TAGCTT_L001_R2.fq.gz |
| PRJNA890236 | SRR22188299 | SRP406242 | SAMN31282243 | Zt120_2227_LIB27072_LDI24051_GGCTAC_L001_R1.fq.gz | Zt120_2227_LIB27072_LDI24051_GGCTAC_L001_R2.fq.gz |

**Raw Data: RNA Sequences**

 SRA links to raw files (forward and reverse) with sample descriptors:

|  | \| [GSM6916835](https://www.ncbi.nlm.nih.gov/geo/query/acc.cgi?acc=GSM6916835) \| In-planta mock infection, 6 days post infection, replicate 1 \| \| --- \| --- \| \| [GSM6916836](https://www.ncbi.nlm.nih.gov/geo/query/acc.cgi?acc=GSM6916836) \| In-planta mock infection, 6 days post infection, replicate 2 \| \| [GSM6916837](https://www.ncbi.nlm.nih.gov/geo/query/acc.cgi?acc=GSM6916837) \| In-planta mock infection, 6 days post infection, replicate 4 \| \| [GSM6916838](https://www.ncbi.nlm.nih.gov/geo/query/acc.cgi?acc=GSM6916838) \| In-planta mock infection, 9 days post infection, replicate 1 \| \| \| [GSM6916839](https://www.ncbi.nlm.nih.gov/geo/query/acc.cgi?acc=GSM6916839) \| In-planta mock infection, 9 days post infection, replicate 2 \| \| \| [GSM6916840](https://www.ncbi.nlm.nih.gov/geo/query/acc.cgi?acc=GSM6916840) \| In-planta mock infection, 9 days post infection, replicate 3 \| \| \| [GSM6916841](https://www.ncbi.nlm.nih.gov/geo/query/acc.cgi?acc=GSM6916841) \| In-planta Z.tritici strain Zt114 infection, 6 days post infection, replicate 1 \| \| \| [GSM6916842](https://www.ncbi.nlm.nih.gov/geo/query/acc.cgi?acc=GSM6916842) \| In-planta Z.tritici strain Zt114 infection, 6 days post infection, replicate 2 \| \| \| [GSM6916843](https://www.ncbi.nlm.nih.gov/geo/query/acc.cgi?acc=GSM6916843) \| In-planta Z.tritici strain Zt114 infection, 6 days post infection, replicate 3 \| \| \| [GSM6916844](https://www.ncbi.nlm.nih.gov/geo/query/acc.cgi?acc=GSM6916844) \| In-planta Z.tritici strain Zt114 infection, 9 days post infection, replicate 1 \| \| \| [GSM6916845](https://www.ncbi.nlm.nih.gov/geo/query/acc.cgi?acc=GSM6916845) \| In-planta Z.tritici strain Zt114 infection, 9 days post infection, replicate 2 \| \| \| [GSM6916846](https://www.ncbi.nlm.nih.gov/geo/query/acc.cgi?acc=GSM6916846) \| In-planta Z.tritici strain Zt114 infection, 9 days post infection, replicate 3 \| \| \| [GSM6916847](https://www.ncbi.nlm.nih.gov/geo/query/acc.cgi?acc=GSM6916847) \| In-vitro Z.tritici strain Zt114, replicate 1 \| \| \| [GSM6916848](https://www.ncbi.nlm.nih.gov/geo/query/acc.cgi?acc=GSM6916848) \| In-vitro Z.tritici strain Zt114, replicate 2 \| \| \| [GSM6916849](https://www.ncbi.nlm.nih.gov/geo/query/acc.cgi?acc=GSM6916849) \| In-vitro Z.tritici strain Zt114, replicate 3 \| \| \| [GSM6916850](https://www.ncbi.nlm.nih.gov/geo/query/acc.cgi?acc=GSM6916850) \| In-planta Z.tritici strain Zt116 infection, 6 days post infection, replicate 1 \| \| \| [GSM6916851](https://www.ncbi.nlm.nih.gov/geo/query/acc.cgi?acc=GSM6916851) \| In-planta Z.tritici strain Zt116 infection, 6 days post infection, replicate 2 \| \| \| [GSM6916852](https://www.ncbi.nlm.nih.gov/geo/query/acc.cgi?acc=GSM6916852) \| In-planta Z.tritici strain Zt116 infection, 6 days post infection, replicate 3 \| \| \| [GSM6916853](https://www.ncbi.nlm.nih.gov/geo/query/acc.cgi?acc=GSM6916853) \| In-planta Z.tritici strain Zt116 infection, 9 days post infection, replicate 1 \| \| \| [GSM6916854](https://www.ncbi.nlm.nih.gov/geo/query/acc.cgi?acc=GSM6916854) \| In-planta Z.tritici strain Zt116 infection, 9 days post infection, replicate 2 \| \| \| [GSM6916855](https://www.ncbi.nlm.nih.gov/geo/query/acc.cgi?acc=GSM6916855) \| In-planta Z.tritici strain Zt116 infection, 9 days post infection, replicate 4 \| \| \| [GSM6916856](https://www.ncbi.nlm.nih.gov/geo/query/acc.cgi?acc=GSM6916856) \| In-vitro Z.tritici strain Zt116, replicate 1 \| \| \| [GSM6916857](https://www.ncbi.nlm.nih.gov/geo/query/acc.cgi?acc=GSM6916857) \| In-vitro Z.tritici strain Zt116, replicate 2 \| \| \| [GSM6916858](https://www.ncbi.nlm.nih.gov/geo/query/acc.cgi?acc=GSM6916858) \| In-vitro Z.tritici strain Zt116, replicate 3 \| \| \| [GSM6916859](https://www.ncbi.nlm.nih.gov/geo/query/acc.cgi?acc=GSM6916859) \| In-planta Z.tritici strain Zt117 infection, 6 days post infection, replicate 2 \| \| \| [GSM6916860](https://www.ncbi.nlm.nih.gov/geo/query/acc.cgi?acc=GSM6916860) \| In-planta Z.tritici strain Zt117 infection, 6 days post infection, replicate 3 \| \| \| [GSM6916861](https://www.ncbi.nlm.nih.gov/geo/query/acc.cgi?acc=GSM6916861) \| In-planta Z.tritici strain Zt117 infection, 6 days post infection, replicate 4 \| \| \| [GSM6916862](https://www.ncbi.nlm.nih.gov/geo/query/acc.cgi?acc=GSM6916862) \| In-planta Z.tritici strain Zt117 infection, 9 days post infection, replicate 1 \| \| \| [GSM6916863](https://www.ncbi.nlm.nih.gov/geo/query/acc.cgi?acc=GSM6916863) \| In-planta Z.tritici strain Zt117 infection, 9 days post infection, replicate 2 \| \| \| [GSM6916864](https://www.ncbi.nlm.nih.gov/geo/query/acc.cgi?acc=GSM6916864) \| In-planta Z.tritici strain Zt117 infection, 9 days post infection, replicate 4 \| \| \| [GSM6916865](https://www.ncbi.nlm.nih.gov/geo/query/acc.cgi?acc=GSM6916865) \| In-vitro Z.tritici strain Zt117, replicate 1 \| \| \| [GSM6916866](https://www.ncbi.nlm.nih.gov/geo/query/acc.cgi?acc=GSM6916866) \| In-vitro Z.tritici strain Zt117, replicate 2 \| \| \| [GSM6916867](https://www.ncbi.nlm.nih.gov/geo/query/acc.cgi?acc=GSM6916867) \| In-vitro Z.tritici strain Zt117, replicate 3 \| \| \| [GSM6916868](https://www.ncbi.nlm.nih.gov/geo/query/acc.cgi?acc=GSM6916868) \| In-planta Z.tritici strain Zt118 infection, 6 days post infection, replicate 1 \| \| \| [GSM6916869](https://www.ncbi.nlm.nih.gov/geo/query/acc.cgi?acc=GSM6916869) \| In-planta Z.tritici strain Zt118 infection, 6 days post infection, replicate 2 \| \| \| [GSM6916870](https://www.ncbi.nlm.nih.gov/geo/query/acc.cgi?acc=GSM6916870) \| In-planta Z.tritici strain Zt118 infection, 6 days post infection, replicate 4 \| \| \| [GSM6916871](https://www.ncbi.nlm.nih.gov/geo/query/acc.cgi?acc=GSM6916871) \| In-planta Z.tritici strain Zt118 infection, 9 days post infection, replicate 1 \| \| \| [GSM6916872](https://www.ncbi.nlm.nih.gov/geo/query/acc.cgi?acc=GSM6916872) \| In-planta Z.tritici strain Zt118 infection, 9 days post infection, replicate 2 \| \| \| [GSM6916873](https://www.ncbi.nlm.nih.gov/geo/query/acc.cgi?acc=GSM6916873) \| In-planta Z.tritici strain Zt118 infection, 9 days post infection, replicate 3 \| \| \| [GSM6916874](https://www.ncbi.nlm.nih.gov/geo/query/acc.cgi?acc=GSM6916874) \| In-vitro Z.tritici strain Zt118, replicate 1 \| \| \| [GSM6916875](https://www.ncbi.nlm.nih.gov/geo/query/acc.cgi?acc=GSM6916875) \| In-vitro Z.tritici strain Zt118, replicate 2 \| \| \| [GSM6916876](https://www.ncbi.nlm.nih.gov/geo/query/acc.cgi?acc=GSM6916876) \| In-vitro Z.tritici strain Zt118, replicate 3 \| \| \| [GSM6916877](https://www.ncbi.nlm.nih.gov/geo/query/acc.cgi?acc=GSM6916877) \| In-planta Z.tritici strain Zt36 infection, 6 days post infection, replicate 2 \| \| \| [GSM6916878](https://www.ncbi.nlm.nih.gov/geo/query/acc.cgi?acc=GSM6916878) \| In-planta Z.tritici strain Zt36 infection, 6 days post infection, replicate 3 \| \| \| [GSM6916879](https://www.ncbi.nlm.nih.gov/geo/query/acc.cgi?acc=GSM6916879) \| In-planta Z.tritici strain Zt36 infection, 6 days post infection, replicate 4 \| \| \| [GSM6916880](https://www.ncbi.nlm.nih.gov/geo/query/acc.cgi?acc=GSM6916880) \| In-planta Z.tritici strain Zt36 infection, 9 days post infection, replicate 1 \| \| \| [GSM6916881](https://www.ncbi.nlm.nih.gov/geo/query/acc.cgi?acc=GSM6916881) \| In-planta Z.tritici strain Zt36 infection, 9 days post infection, replicate 3 \| \| \| [GSM6916882](https://www.ncbi.nlm.nih.gov/geo/query/acc.cgi?acc=GSM6916882) \| In-planta Z.tritici strain Zt36 infection, 9 days post infection, replicate 4 \| \| \| [GSM6916883](https://www.ncbi.nlm.nih.gov/geo/query/acc.cgi?acc=GSM6916883) \| In-vitro Z.tritici strain Zt36, replicate 1 \| \| \| [GSM6916884](https://www.ncbi.nlm.nih.gov/geo/query/acc.cgi?acc=GSM6916884) \| In-vitro Z.tritici strain Zt36, replicate 2 \| \| \| [GSM6916885](https://www.ncbi.nlm.nih.gov/geo/query/acc.cgi?acc=GSM6916885) \| In-vitro Z.tritici strain Zt36, replicate 3 \| \| \| [GSM6916886](https://www.ncbi.nlm.nih.gov/geo/query/acc.cgi?acc=GSM6916886) \| In-planta Z.tritici strain Zt48 infection, 6 days post infection, replicate 1 \| \| \| [GSM6916887](https://www.ncbi.nlm.nih.gov/geo/query/acc.cgi?acc=GSM6916887) \| In-planta Z.tritici strain Zt48 infection, 6 days post infection, replicate 2 \| \| \| [GSM6916888](https://www.ncbi.nlm.nih.gov/geo/query/acc.cgi?acc=GSM6916888) \| In-planta Z.tritici strain Zt48 infection, 6 days post infection, replicate 3 \| \| \| [GSM6916889](https://www.ncbi.nlm.nih.gov/geo/query/acc.cgi?acc=GSM6916889) \| In-planta Z.tritici strain Zt48 infection, 9 days post infection, replicate 1 \| \| \| [GSM6916890](https://www.ncbi.nlm.nih.gov/geo/query/acc.cgi?acc=GSM6916890) \| In-planta Z.tritici strain Zt48 infection, 9 days post infection, replicate 2 \| \| \| [GSM6916891](https://www.ncbi.nlm.nih.gov/geo/query/acc.cgi?acc=GSM6916891) \| In-planta Z.tritici strain Zt48 infection, 9 days post infection, replicate 4 \| \| \| [GSM6916892](https://www.ncbi.nlm.nih.gov/geo/query/acc.cgi?acc=GSM6916892) \| In-vitro Z.tritici strain Zt48, replicate 1 \| \| \| [GSM6916893](https://www.ncbi.nlm.nih.gov/geo/query/acc.cgi?acc=GSM6916893) \| In-vitro Z.tritici strain Zt48, replicate 2 \| \| \| [GSM6916894](https://www.ncbi.nlm.nih.gov/geo/query/acc.cgi?acc=GSM6916894) \| In-vitro Z.tritici strain Zt48, replicate 3 \| \| \| [GSM6916895](https://www.ncbi.nlm.nih.gov/geo/query/acc.cgi?acc=GSM6916895) \| In-planta Z.tritici strain Zt74 infection, 6 days post infection, replicate 1 \| \| \| [GSM6916896](https://www.ncbi.nlm.nih.gov/geo/query/acc.cgi?acc=GSM6916896) \| In-planta Z.tritici strain Zt74 infection, 6 days post infection, replicate 2 \| \| \| [GSM6916897](https://www.ncbi.nlm.nih.gov/geo/query/acc.cgi?acc=GSM6916897) \| In-planta Z.tritici strain Zt74 infection, 6 days post infection, replicate 3 \| \| \| [GSM6916898](https://www.ncbi.nlm.nih.gov/geo/query/acc.cgi?acc=GSM6916898) \| In-planta Z.tritici strain Zt74 infection, 9 days post infection, replicate 1 \| \| \| [GSM6916899](https://www.ncbi.nlm.nih.gov/geo/query/acc.cgi?acc=GSM6916899) \| In-planta Z.tritici strain Zt74 infection, 9 days post infection, replicate 3 \| \| \| [GSM6916900](https://www.ncbi.nlm.nih.gov/geo/query/acc.cgi?acc=GSM6916900) \| In-planta Z.tritici strain Zt74 infection, 9 days post infection, replicate 4 \| \| \| [GSM6916901](https://www.ncbi.nlm.nih.gov/geo/query/acc.cgi?acc=GSM6916901) \| In-vitro Z.tritici strain Zt74, replicate 1 \| \| \| [GSM6916902](https://www.ncbi.nlm.nih.gov/geo/query/acc.cgi?acc=GSM6916902) \| In-vitro Z.tritici strain Zt74, replicate 2 \| \| \| [GSM6916903](https://www.ncbi.nlm.nih.gov/geo/query/acc.cgi?acc=GSM6916903) \| In-vitro Z.tritici strain Zt74, replicate 3 \| \| \| [GSM6916904](https://www.ncbi.nlm.nih.gov/geo/query/acc.cgi?acc=GSM6916904) \| In-planta Z.tritici strain Zt80 infection, 6 days post infection, replicate 1 \| \| \| [GSM6916905](https://www.ncbi.nlm.nih.gov/geo/query/acc.cgi?acc=GSM6916905) \| In-planta Z.tritici strain Zt80 infection, 6 days post infection, replicate 3 \| \| \| [GSM6916906](https://www.ncbi.nlm.nih.gov/geo/query/acc.cgi?acc=GSM6916906) \| In-planta Z.tritici strain Zt80 infection, 6 days post infection, replicate 4 \| \| \| [GSM6916907](https://www.ncbi.nlm.nih.gov/geo/query/acc.cgi?acc=GSM6916907) \| In-planta Z.tritici strain Zt80 infection, 9 days post infection, replicate 2 \| \| \| [GSM6916908](https://www.ncbi.nlm.nih.gov/geo/query/acc.cgi?acc=GSM6916908) \| In-planta Z.tritici strain Zt80 infection, 9 days post infection, replicate 3 \| \| \| [GSM6916909](https://www.ncbi.nlm.nih.gov/geo/query/acc.cgi?acc=GSM6916909) \| In-planta Z.tritici strain Zt80 infection, 9 days post infection, replicate 4 \| \| \| [GSM6916910](https://www.ncbi.nlm.nih.gov/geo/query/acc.cgi?acc=GSM6916910) \| In-vitro Z.tritici strain Zt80, replicate 1 \| \| \| [GSM6916911](https://www.ncbi.nlm.nih.gov/geo/query/acc.cgi?acc=GSM6916911) \| In-vitro Z.tritici strain Zt80, replicate 2 \| \| \| [GSM6916912](https://www.ncbi.nlm.nih.gov/geo/query/acc.cgi?acc=GSM6916912) \| In-vitro Z.tritici strain Zt80, replicate 3 \| \| \| [GSM6916913](https://www.ncbi.nlm.nih.gov/geo/query/acc.cgi?acc=GSM6916913) \| In-planta Z.tritici strain Zt88 infection, 6 days post infection, replicate 1 \| \| \| [GSM6916914](https://www.ncbi.nlm.nih.gov/geo/query/acc.cgi?acc=GSM6916914) \| In-planta Z.tritici strain Zt88 infection, 6 days post infection, replicate 2 \| \| \| [GSM6916915](https://www.ncbi.nlm.nih.gov/geo/query/acc.cgi?acc=GSM6916915) \| In-planta Z.tritici strain Zt88 infection, 6 days post infection, replicate 4 \| \| \| [GSM6916916](https://www.ncbi.nlm.nih.gov/geo/query/acc.cgi?acc=GSM6916916) \| In-planta Z.tritici strain Zt88 infection, 9 days post infection, replicate 2 \| \| \| [GSM6916917](https://www.ncbi.nlm.nih.gov/geo/query/acc.cgi?acc=GSM6916917) \| In-planta Z.tritici strain Zt88 infection, 9 days post infection, replicate 3 \| \| \| [GSM6916918](https://www.ncbi.nlm.nih.gov/geo/query/acc.cgi?acc=GSM6916918) \| In-planta Z.tritici strain Zt88 infection, 9 days post infection, replicate 4 \| \| \| [GSM6916919](https://www.ncbi.nlm.nih.gov/geo/query/acc.cgi?acc=GSM6916919) \| In-vitro Z.tritici strain Zt88, replicate 1 \| \| \| [GSM6916920](https://www.ncbi.nlm.nih.gov/geo/query/acc.cgi?acc=GSM6916920) \| In-vitro Z.tritici strain Zt88, replicate 2 \| \| \| [GSM6916921](https://www.ncbi.nlm.nih.gov/geo/query/acc.cgi?acc=GSM6916921) \| In-vitro Z.tritici strain Zt88, replicate 3 \| \| \| [GSM6916922](https://www.ncbi.nlm.nih.gov/geo/query/acc.cgi?acc=GSM6916922) \| In-planta Z.tritici strain Zt92 infection, 6 days post infection, replicate 1 \| \| \| [GSM6916923](https://www.ncbi.nlm.nih.gov/geo/query/acc.cgi?acc=GSM6916923) \| In-planta Z.tritici strain Zt92 infection, 6 days post infection, replicate 2 \| \| \| [GSM6916924](https://www.ncbi.nlm.nih.gov/geo/query/acc.cgi?acc=GSM6916924) \| In-planta Z.tritici strain Zt92 infection, 6 days post infection, replicate 3 \| \| \| [GSM6916925](https://www.ncbi.nlm.nih.gov/geo/query/acc.cgi?acc=GSM6916925) \| In-planta Z.tritici strain Zt92 infection, 9 days post infection, replicate 1 \| \| \| [GSM6916926](https://www.ncbi.nlm.nih.gov/geo/query/acc.cgi?acc=GSM6916926) \| In-planta Z.tritici strain Zt92 infection, 9 days post infection, replicate 2 \| \| \| [GSM6916927](https://www.ncbi.nlm.nih.gov/geo/query/acc.cgi?acc=GSM6916927) \| In-planta Z.tritici strain Zt92 infection, 9 days post infection, replicate 4 \| \| \| [GSM6916928](https://www.ncbi.nlm.nih.gov/geo/query/acc.cgi?acc=GSM6916928) \| In-vitro Z.tritici strain Zt92, replicate 1 \| \| \| [GSM6916929](https://www.ncbi.nlm.nih.gov/geo/query/acc.cgi?acc=GSM6916929) \| In-vitro Z.tritici strain Zt92, replicate 2 \| \| \| [GSM6916930](https://www.ncbi.nlm.nih.gov/geo/query/acc.cgi?acc=GSM6916930) \| In-vitro Z.tritici strain Zt92, replicate 3 \| \| \| [GSM6916931](https://www.ncbi.nlm.nih.gov/geo/query/acc.cgi?acc=GSM6916931) \| In-planta Z.tritici strain Zt93 infection, 6 days post infection, replicate 2 \| \| \| [GSM6916932](https://www.ncbi.nlm.nih.gov/geo/query/acc.cgi?acc=GSM6916932) \| In-planta Z.tritici strain Zt93 infection, 6 days post infection, replicate 3 \| \| \| [GSM6916933](https://www.ncbi.nlm.nih.gov/geo/query/acc.cgi?acc=GSM6916933) \| In-planta Z.tritici strain Zt93 infection, 6 days post infection, replicate 4 \| \| \| [GSM6916934](https://www.ncbi.nlm.nih.gov/geo/query/acc.cgi?acc=GSM6916934) \| In-planta Z.tritici strain Zt93 infection, 9 days post infection, replicate 2 \| \| \| [GSM6916935](https://www.ncbi.nlm.nih.gov/geo/query/acc.cgi?acc=GSM6916935) \| In-planta Z.tritici strain Zt93 infection, 9 days post infection, replicate 3 \| \| \| [GSM6916936](https://www.ncbi.nlm.nih.gov/geo/query/acc.cgi?acc=GSM6916936) \| In-planta Z.tritici strain Zt93 infection, 9 days post infection, replicate 4 \| \| \| [GSM6916937](https://www.ncbi.nlm.nih.gov/geo/query/acc.cgi?acc=GSM6916937) \| In-vitro Z.tritici strain Zt93, replicate 1 \| \| \| [GSM6916938](https://www.ncbi.nlm.nih.gov/geo/query/acc.cgi?acc=GSM6916938) \| In-vitro Z.tritici strain Zt93, replicate 2 \| \| \| [GSM6916939](https://www.ncbi.nlm.nih.gov/geo/query/acc.cgi?acc=GSM6916939) \| In-vitro Z.tritici strain Zt93, replicate 3 \| \| \| [GSM6916940](https://www.ncbi.nlm.nih.gov/geo/query/acc.cgi?acc=GSM6916940) \| In-planta Z.tritici strain Zt99 infection, 6 days post infection, replicate 2 \| \| \| [GSM6916941](https://www.ncbi.nlm.nih.gov/geo/query/acc.cgi?acc=GSM6916941) \| In-planta Z.tritici strain Zt99 infection, 6 days post infection, replicate 3 \| \| \| [GSM6916942](https://www.ncbi.nlm.nih.gov/geo/query/acc.cgi?acc=GSM6916942) \| In-planta Z.tritici strain Zt99 infection, 6 days post infection, replicate 4 \| \| \| [GSM6916943](https://www.ncbi.nlm.nih.gov/geo/query/acc.cgi?acc=GSM6916943) \| In-planta Z.tritici strain Zt99 infection, 9 days post infection, replicate 1 \| \| \| [GSM6916944](https://www.ncbi.nlm.nih.gov/geo/query/acc.cgi?acc=GSM6916944) \| In-planta Z.tritici strain Zt99 infection, 9 days post infection, replicate 3 \| \| \| [GSM6916945](https://www.ncbi.nlm.nih.gov/geo/query/acc.cgi?acc=GSM6916945) \| In-planta Z.tritici strain Zt99 infection, 9 days post infection, replicate 4 \| \| \| [GSM6916946](https://www.ncbi.nlm.nih.gov/geo/query/acc.cgi?acc=GSM6916946) \| In-vitro Z.tritici strain Zt99, replicate 1 \| \| \| [GSM6916947](https://www.ncbi.nlm.nih.gov/geo/query/acc.cgi?acc=GSM6916947) \| In-vitro Z.tritici strain Zt99, replicate 2 \| \| \| [GSM6916948](https://www.ncbi.nlm.nih.gov/geo/query/acc.cgi?acc=GSM6916948) \| In-vitro Z.tritici strain Zt99, replicate 3 \| \| |
| --- | --- | --- | --- | --- | --- | --- | --- | --- | --- | --- | --- | --- | --- | --- | --- | --- | --- | --- | --- | --- | --- | --- | --- | --- | --- | --- | --- | --- | --- | --- | --- | --- | --- | --- | --- | --- | --- | --- | --- | --- | --- | --- | --- | --- | --- | --- | --- | --- | --- | --- | --- | --- | --- | --- | --- | --- | --- | --- | --- | --- | --- | --- | --- | --- | --- | --- | --- | --- | --- | --- | --- | --- | --- | --- | --- | --- | --- | --- | --- | --- | --- | --- | --- | --- | --- | --- | --- | --- | --- | --- | --- | --- | --- | --- | --- | --- | --- | --- | --- | --- | --- | --- | --- | --- | --- | --- | --- | --- | --- | --- | --- | --- | --- | --- | --- | --- | --- | --- | --- | --- | --- | --- | --- | --- | --- | --- | --- | --- | --- | --- | --- | --- | --- | --- | --- | --- | --- | --- | --- | --- | --- | --- | --- | --- | --- | --- | --- | --- | --- | --- | --- | --- | --- | --- | --- | --- | --- | --- | --- | --- | --- | --- | --- | --- | --- | --- | --- | --- | --- | --- | --- | --- | --- | --- | --- | --- | --- | --- | --- | --- | --- | --- | --- | --- | --- | --- | --- | --- | --- | --- | --- | --- | --- | --- | --- | --- | --- | --- | --- | --- | --- | --- | --- | --- | --- | --- | --- | --- | --- | --- | --- | --- | --- | --- | --- | --- | --- | --- | --- | --- | --- | --- | --- | --- | --- | --- | --- | --- | --- | --- | --- | --- | --- | --- | --- | --- | --- | --- | --- | --- | --- | --- | --- | --- | --- | --- | --- | --- | --- | --- | --- | --- | --- | --- | --- | --- | --- | --- | --- | --- | --- | --- | --- | --- | --- | --- | --- | --- | --- | --- | --- | --- | --- | --- | --- | --- | --- | --- | --- | --- | --- | --- | --- | --- | --- | --- | --- | --- | --- | --- | --- | --- | --- | --- | --- | --- | --- | --- | --- | --- | --- | --- | --- | --- | --- | --- | --- | --- | --- | --- | --- | --- | --- | --- | --- | --- | --- | --- | --- | --- | --- | --- | --- | --- | --- | --- | --- | --- | --- | --- | --- | --- | --- | --- | --- | --- | --- | --- | --- | --- |

**Derived Data: Genome Assemblies**

| NCBI BioProject | NCBI Accession | Strain | Species | NCBI Taxon ID |
| --- | --- | --- | --- | --- |
| PRJNA890236 | <https://www.ncbi.nlm.nih.gov/biosample/SAMN31282227> | Zt10 | Zymoseptoria tritici | 1047171 |
| PRJNA890236 | <https://www.ncbi.nlm.nih.gov/biosample/SAMN31282228> | Zt36 | Zymoseptoria tritici | 1047171 |
| PRJNA890236 | <https://www.ncbi.nlm.nih.gov/biosample/SAMN31282229> | Zt48 | Zymoseptoria tritici | 1047171 |
| PRJNA890236 | <https://www.ncbi.nlm.nih.gov/biosample/SAMN31282230> | Zt55 | Zymoseptoria tritici | 1047171 |
| PRJNA890236 | <https://www.ncbi.nlm.nih.gov/biosample/SAMN31282231> | Zt71 | Zymoseptoria tritici | 1047171 |
| PRJNA890236 | <https://www.ncbi.nlm.nih.gov/biosample/SAMN31282232> | Zt74 | Zymoseptoria tritici | 1047171 |
| PRJNA890236 | <https://www.ncbi.nlm.nih.gov/biosample/SAMN31282233> | Zt80 | Zymoseptoria tritici | 1047171 |
| PRJNA890236 | <https://www.ncbi.nlm.nih.gov/biosample/SAMN31282234> | Zt88 | Zymoseptoria tritici | 1047171 |
| PRJNA890236 | <https://www.ncbi.nlm.nih.gov/biosample/SAMN31282235> | Zt92 | Zymoseptoria tritici | 1047171 |
| PRJNA890236 | <https://www.ncbi.nlm.nih.gov/biosample/SAMN31282236> | Zt93 | Zymoseptoria tritici | 1047171 |
| PRJNA890236 | <https://www.ncbi.nlm.nih.gov/biosample/SAMN31282237> | Zt97 | Zymoseptoria tritici | 1047171 |
| PRJNA890236 | <https://www.ncbi.nlm.nih.gov/biosample/SAMN31282238> | Zt99 | Zymoseptoria tritici | 1047171 |
| PRJNA890236 | <https://www.ncbi.nlm.nih.gov/biosample/SAMN31282239> | Zt114 | Zymoseptoria tritici | 1047171 |
| PRJNA890236 | <https://www.ncbi.nlm.nih.gov/biosample/SAMN31282240> | Zt116 | Zymoseptoria tritici | 1047171 |
| PRJNA890236 | <https://www.ncbi.nlm.nih.gov/biosample/SAMN31282241> | Zt117 | Zymoseptoria tritici | 1047171 |
| PRJNA890236 | <https://www.ncbi.nlm.nih.gov/biosample/SAMN31282242> | Zt118 | Zymoseptoria tritici | 1047171 |
| PRJNA890236 | <https://www.ncbi.nlm.nih.gov/biosample/SAMN31282243> | Zt120 | Zymoseptoria tritici | 1047171 |

**Derived Data: gene sequences and annotation**

The pangene sequences and annotation are submitted with the paper as supplementary files for download and are also available following publication at the Rothamsted repository at <https://doi.org/10.23637/rothamsted.98q90>:

Pangenome_Zt_17I_IPO323_v3.fasta   (also available at NCBI GEO, see below)

annotation_pangenomveV3_2.gtf

**Derived Data: Gene Expression Counts (in-vitro and in-planta)**

| GSE222164_Pangenome_Zt_17I_IPO323_v3.fasta.gz | 12.6 Mb | [(http)](https://www.ncbi.nlm.nih.gov/geo/download/?acc=GSE222164&format=file&file=GSE222164%5FPangenome%5FZt%5F17I%5FIPO323%5Fv3%2Efasta%2Egz) | FASTA |
| --- | --- | --- | --- |
| GSE222164_ztriticiPangenomePlantaCounts.txt.gz | 2.3 Mb | [(http)](https://www.ncbi.nlm.nih.gov/geo/download/?acc=GSE222164&format=file&file=GSE222164%5FztriticiPangenomePlantaCounts%2Etxt%2Egz) | TXT |

Both raw and these derived expression files Accessible from NCBI GEO accession GSE222164 (<https://www.ncbi.nlm.nih.gov/geo/query/acc.cgi?acc=GSE222164> )
